# Comparison of 6q25 Breast Cancer Hits from Asian and European Genome Wide Association Studies in the Breast Cancer Association Consortium (BCAC)

**DOI:** 10.1371/journal.pone.0042380

**Published:** 2012-08-07

**Authors:** Rebecca Hein, Melanie Maranian, John L. Hopper, Miroslaw K. Kapuscinski, Melissa C. Southey, Daniel J. Park, Marjanka K. Schmidt, Annegien Broeks, Frans B. L. Hogervorst, H. Bas Bueno-de-Mesquit, Kenneth R. Muir, Artitaya Lophatananon, Suthee Rattanamongkongul, Puttisak Puttawibul, Peter A. Fasching, Alexander Hein, Arif B. Ekici, Matthias W. Beckmann, Olivia Fletcher, Nichola Johnson, Isabel dos Santos Silva, Julian Peto, Elinor Sawyer, Ian Tomlinson, Michael Kerin, Nicola Miller, Frederick Marmee, Andreas Schneeweiss, Christof Sohn, Barbara Burwinkel, Pascal Guénel, Emilie Cordina-Duverger, Florence Menegaux, Thérèse Truong, Stig E. Bojesen, Børge G. Nordestgaard, Henrik Flyger, Roger L. Milne, Jose Ignacio Arias Perez, M. Pilar Zamora, Javier Benítez, Hoda Anton-Culver, Argyrios Ziogas, Leslie Bernstein, Christina A. Clarke, Hermann Brenner, Heiko Müller, Volker Arndt, Christa Stegmaier, Nazneen Rahman, Sheila Seal, Clare Turnbull, Anthony Renwick, Alfons Meindl, Sarah Schott, Claus R. Bartram, Rita K. Schmutzler, Hiltrud Brauch, Ute Hamann, Yon-Dschun Ko, The GENICA Network, Shan Wang-Gohrke, Thilo Dörk, Peter Schürmann, Johann H. Karstens, Peter Hillemanns, Heli Nevanlinna, Tuomas Heikkinen, Kristiina Aittomäki, Carl Blomqvist, Natalia V. Bogdanova, Iosif V. Zalutsky, Natalia N. Antonenkova, Marina Bermisheva, Darya Prokovieva, Albina Farahtdinova, Elza Khusnutdinova, Annika Lindblom, Sara Margolin, Arto Mannermaa, Vesa Kataja, Veli-Matti Kosma, Jaana Hartikainen, Xiaoqing Chen, Jonathan Beesley, kConFab Investigators, AOCS Group, Diether Lambrechts, Hui Zhao, Patrick Neven, Hans Wildiers, Stefan Nickels, Dieter Flesch-Janys, Paolo Radice, Paolo Peterlongo, Siranoush Manoukian, Monica Barile, Fergus J. Couch, Janet E. Olson, Xianshu Wang, Zachary Fredericksen, Graham G. Giles, Laura Baglietto, Catriona A. McLean, Gianluca Severi, Kenneth Offit, Mark Robson, Mia M. Gaudet, Joseph Vijai, Grethe Grenaker Alnæs, Vessela Kristensen, Anne-Lise Børresen-Dale, Esther M. John, Alexander Miron, Robert Winqvist, Katri Pylkäs, Arja Jukkola-Vuorinen, Mervi Grip, Irene L. Andrulis, Julia A. Knight, Gord Glendon, Anna Marie Mulligan, Jonine D. Figueroa, Montserrat García-Closas, Jolanta Lissowska, Mark E. Sherman, Maartje Hooning, John W. M. Martens, Caroline Seynaeve, Margriet Collée, Per Hall, Keith Humpreys, Kamila Czene, Jianjun Liu, Angela Cox, Ian W. Brock, Simon S. Cross, Malcolm W. R. Reed, Shahana Ahmed, Maya Ghoussaini, Paul DP. Pharoah, Daehee Kang, Keun-Young Yoo, Dong-Young Noh, Anna Jakubowska, Katarzyna Jaworska, Katarzyna Durda, Elżbieta Złowocka, Suleeporn Sangrajrang, Valerie Gaborieau, Paul Brennan, James McKay, Chen-Yang Shen, Jyh-Cherng Yu, Huan-Ming Hsu, Ming-Feng Hou, Nick Orr, Minouk Schoemaker, Alan Ashworth, Anthony Swerdlow, Amy Trentham-Dietz, Polly A. Newcomb, Linda Titus, Kathleen M. Egan, Georgia Chenevix-Trench, Antonis C. Antoniou, Manjeet K. Humphreys, Jonathan Morrison, Jenny Chang-Claude, Douglas F. Easton, Alison M. Dunning

**Affiliations:** 1 Unit of Genetic Epidemiology, Division of Cancer Epidemiology, German Cancer Research Center (DKFZ), Heidelberg, Germany; 2 PMV (Primärmedizinische Versorgung) Research Group at the Department of Child and Adolescent Psychiatry and Psychotherapy, University of Cologne, Cologne, Germany; 3 Department of Oncology, University of Cambridge, Cambridge, United Kingdom; 4 Centre for Molecular, Environmental, Genetic and Analytic Epidemiology, The University of Melbourne, Melbourne, Australia; 5 Department of Pathology, The University of Melbourne, Melbourne, Australia; 6 Genetic Epidemiology Laboratory, Department of Pathology, The University of Melbourne, Melbourne, Australia; 7 Netherlands Cancer Institute, Antoni van Leeuwenhoek hospital, Amsterdam, The Netherlands; 8 Family Cancer Clinic, Netherlands Cancer Institute, Antoni van Leeuwenhoek Hospital, Amsterdam, The Netherlands; 9 National Institute for Public Health and the Environment, Bilthoven, The Netherlands; 10 Health Sciences Research Institute, Warwick Medical School, Warwick University, Coventry, Warwick, United Kingdom; 11 Department of Preventive Medicine, Srinakhrainwirot University, Ongkharak, Nakhon Nayok, Thailand; 12 Department of Surgery, Medical School, Prince Songkla University, Songkla, Thailand; 13 University Breast Center, Department of Gynecology and Obstetrics, University Hospital Erlangen, Erlangen, Germany; 14 David Geffen School of Medicine, Department of Medicine Division of Hematology and Oncology, University of California at Los Angeles, Los Angeles, California, United States of America; 15 Institute of Human Genetics, Friedrich Alexander University Erlangen-Nuremberg, Erlangen, Germany; 16 Breakthrough Breast Cancer Research Centre, The Institute of Cancer Research, London, United Kingdom; 17 Department of Non-Communicable Disease Epidemiology, London School of Hygiene and Tropical Medicine, London, United Kingdom; 18 Division of Cancer Studies, National Institute for Health Research Comprehensive Biomedical Research Centre, Guy’s & St. Thomas’ National Health Service Foundation Trust in partnership with King’s College London, London, United Kingdom; 19 Welcome Trust Centre for Human Genetics and Oxford Biomedical Research Centre, University of Oxford, Oxford, United Kingdom; 20 Oxford National Institute for Health Research Comprehensive Biomedical Research Centre, Oxford, United Kingdom; 21 Clinical Science Institute. University Hospital Galway, Galway, Ireland; 22 Department of Obstetrics and Gynecology, University of Heidelberg, Heidelberg, Germany; 23 National Center for Tumor Diseases, University of Heidelberg, Heidelberg, Germany; 24 Molecular Epidemiology Group, German Cancer Research Center (DKFZ), Heidelberg, Germany; 25 Environmental Epidemiology of Cancer, U1018, CESP (Center for Research in Epidemiology and Population Health), Inserm (National Institute of Health and Medical Research), Villejuif, France; 26 Unité mixte de recherche 1018, University Paris-Sud, Villejuif, France; 27 Copenhagen General Population Study and Department of Clinical Biochemistry, Herlev University Hospital, University of Copenhagen, Copenhagen, Denmark; 28 Department of Breast Surgery, Herlev University Hospital, University of Copenhagen, Copenhagen, Denmark; 29 Genetic & Molecular Epidemiology Group, Spanish National Cancer Research Centre (CNIO), Madrid, Spain; 30 Servicio de Cirugía General y Especialidades, Hospital Monte Naranco, Oviedo, Spain; 31 Servicio de Oncología Médica, Hospital Universitario La Paz, Madrid, Spain; 32 Cancer Genetics Group, Spanish National Cancer Research Centre (CNIO), Madrid, Spain; 33 Department of Epidemiology, University of California Irvine, Irvine, California, United States of America; 34 Department of Population Sciences, City of Hope, Duarte, California, United States of America; 35 Cancer Prevention Institute of California, Fremont, California, United States of America; 36 Division of Clinical Epidemiology and Aging Research, German Cancer Research Center, Heidelberg, Germany; 37 Saarland Cancer Registry, Saarbrücken, Germany; 38 Section of Cancer Genetics, Institute of Cancer Research, Sutton, Surrey, United Kingdom; 39 Division of Gynecology and Obstetrics, Klinikum rechts der Isar at the Technical University Munich, Munich, Germany; 40 Department of Obstetrics and Gynecology, University of Heidelberg, Heidelberg, Germany; 41 Institute of Human Genetics, University of Heidelberg, Heidelberg, Germany; 42 Center for Familial Breast and Ovarian Cancer and Center of Integrated Oncology (CIO), University Hospital, Cologne, Germany; 43 Dr. Margarete Fischer-Bosch-Institute of Clinical Pharmacology, Stuttgart, University of Tübingen, Tübingen, Germany; 44 Molecular Genetics of Breast Cancer, Deutsches Krebsforschungszentrum (DKFZ), Heidelberg, Germany; 45 Department of Internal Medicine, Evangelische Kliniken Bonn gGmbH, Johanniter Krankenhaus, Bonn, Germany; 46 Institute of Pathology, University of Bonn, Bonn, Germany; 47 Institute for Prevention and Occupational Medicine of the German Social Accident Insurance (IPA), Bochum, Germany; 48 Department of Obstetrics and Gynecology, University of Ulm, Ulm, Germany; 49 Clinics of Obstetrics and Gynaecology, Hannover Medical School, Hannover, Germany; 50 Clinics of Radiation Oncology, Hannover Medical School, Hannover, Germany; 51 Department of Obstetrics and Gynecology, University of Helsinki and Helsinki University Central Hospital, Helsinki, Finland; 52 Department of Clinical Genetics, Helsinki University Central Hospital, Helsinki, Finland; 53 Department of Oncology, Helsinki University Central Hospital, Helsinki, Finland; 54 Clinics of Obstetrics and Gynaecology, Hannover Medical School, Hannover, Germany, Clinics of Radiation Oncology, Hannover Medical School, Hannover, Germany; 55 N.N. Alexandrov Research Institute of Oncology and Medical Radiology, Minsk, Belarus; 56 Institute of Biochemistry and Genetics, Ufa Scientific Center of Russian Academy of Sciences, Ufa, Russia; 57 Department of Molecular Medicine and Surgery, Karolinska Institutet, Stockholm, Sweden; 58 Department of Oncology Pathology, Karolinska Institutet, Stockholm, Sweden; 59 School of Medicine, Institute of Clinical Medicine, Pathology and Forensic Medicine, University of Eastern Finland, Kuopio, Finland; 60 Biocenter Kuopio, Kuopio University Hospital, Kuopio, Finland; 61 Department of Clinical Pathology, Kuopio University Hospital, Kuopio, Finland; 62 School of Medicine, Institute of Clinical Medicine, Oncology, University of Eastern Finland, Kuopio, Finland; 63 Department of Oncology, Kuopio University Hospital, Kuopio, Finland; 64 Cancer Division, Queensland Institute of Medical Research, Brisbane, Australia; 65 Kathleen Cuningham Foundation Consortium for Research into Familial Breast Cancer [kConFab], Peter MacCallum Cancer Center, Melbourne, Australia; 66 Australian Ovarian Cancer Study [AOCS], Peter MacCallum Cancer Center, Melbourne, Australia; 67 Vesalius Research Center (VRC), Vesalius Research Center, Leuven, Belgium; Vesalius Research Center (VRC), Katholieke Universiteit (KU) Leuven, Leuven, Belgium; 68 Multidisciplinary Breast Center, University Hospital Gasthuisberg, Leuven, Belgium; 69 Department of Cancer Epidemiology/Clinical Cancer Registry and Institute for Medical Biometrics and Epidemiology, University Clinic Hamburg-Eppendorf, Hamburg, Germany; 70 Unit of Molecular Bases of Genetic Risk and Genetic Testing, Department of Preventive and Predictive Medicine, Fondazione IRCCS Istituto Nazionale Tumori (INT), Milan, Italy; 71 Fondazione Istituto FIRC (Fondazione Italiana per la Ricerca sul Cancro) Di Oncologia Molecolare (IFOM), Milan, Italy; 72 Unit of Medical Genetics, Department of Preventive and Predictive Medicine, Fondazione Instituto di Recuvero e Cura a Carattere Scientifico (IRCCS) Istituto Nazionale Tumori (INT), Milan, Italy; 73 Division of Cancer Prevention and Genetics, Istituto Europeo di Oncologia (IEO), Milan, Italy; 74 Department of Laboratory Medicine and Pathology, Mayo Clinic, Rochester, Minnesota, United States of America; 75 Department of Health Sciences Research, Mayo Clinic, Rochester, Minnesota, United States of America; 76 Cancer Epidemiology Centre, Cancer Council Victoria, Melbourne, Australia; 77 Centre for Molecular, Environmental, Genetic, and Analytic Epidemiology, The University of Melbourne, Melbourne, Australia; 78 Department of Anatomical Pathology, The Alfred Hospital, Melbourne, Australia; 79 Clinical Genetics Service, Dept. of Medicine and Cancer Biology and Genetics, Memorial Sloan-Kettering Cancer Center, New York, New York, United States of America; 80 Clinical Genetics Service, Dept. of Medicine, Memorial Sloan-Kettering Cancer Center, New York, New York, United States of America; 81 Epidemiology Research Program, American Cancer Society, Atlanta, Georgia, United States of America; 82 Department of Genetics, Institute for Cancer Research, Oslo University Hospital, Radiumhospitalet, Oslo, Norway; 83 Faculty of Medicine (Faculty Division Ahus), University of Oslo, Oslo, Norway; 84 Department of Epidemiology, Cancer Prevention Institute of California, Fremont, California, United States of America; 85 Stanford University School of Medicine, Stanford, California, United States of America; 86 Deptartment of Cancer Biology, Dana-Farber Cancer Institute, Boston, Massachusetts, United States of America; 87 Laboratory of Cancer Genetics, Department of Clinical Genetics and Biocenter Oulu, University of Oulu, Oulu University Hospital, Oulu, Finland; 88 Department of Oncology, Oulu University Hospital, University of Oulu, Oulu, Finland; 89 Department of Surgery, Oulu University Hospital, University of Oulu, Oulu, Finland; 90 Ontario Cancer Genetics Network, Cancer Care Ontario, Toronto, Ontario, Canada; 91 Fred A. Litwin Center for Cancer Genetics, Samuel Lunenfeld Research Institute, Mount Sinai Hospital, Toronto, Ontario, Canada; 92 Department of Molecular Genetics, University of Toronto, Toronto, Ontario, Canada; 93 Samuel Lunenfeld Research Institute, Mount Sinai Hospital, Toronto, Ontario, Canada; 94 Division of Epidemiology, Dalla Lana School of Public Health, University of Toronto, Toronto, Ontario, Canada; 95 Department of Laboratory Medicine and Pathobiology, University of Toronto, Toronto, Ontario, Canada; 96 Department of Laboratory Medicine, and the Keenan Research Centre of the Li Ka Shing Knowledge Institute, St Michael’s Hospital, Toronto, Ontario, Canada; 97 Division of Cancer Epidemiology and Genetics, National Cancer Institute, Rockville, Maryland, United States of America; 98 Sections of Epidemiology and Genetics, Institute of Cancer Research and Breakthrough Breast Cancer Research Centre, London, United Kingdom; 99 Department of Cancer Epidemiology and Prevention, M. Sklodowska-Curie Memorial Cancer Center & Institute of Oncology, Warsaw, Poland; 100 Department of Medical Oncology, Family Cancer Clinic, Erasmus University Medical Center, Rotterdam, The Netherlands; 101 Department of Medical Oncology, Josephine Nefkens Institute, Erasmus University Medical Center, Rotterdam, The Netherlands; 102 Department of Clinical Genetics, Family Cancer Clinic, Erasmus University Medical Center, Rotterdam, The Netherlands; 103 Department of Medical Epidemiology and Biostatistics, Karolinska Institutet, Stockholm, Sweden; 104 Population Genetics, Genome Institute of Singapore, Singapore, Republic of Singapore; 105 Institute for Cancer Studies, Department of Oncology, University of Sheffield, Sheffield, United Kingdom; 106 Academic Unit of Pathology, Department of Neuroscience, University of Sheffield, Sheffield, United Kingdom; 107 Academic Unit of Surgical Oncology, Department of Oncology, University of Sheffield, Sheffield, United Kingdom; 108 Department of Public Health and Primary Care, University of Cambridge, Cambridge, United Kingdom; 109 Department of Preventive Medicine, Seoul National University College of Medicine, Seoul, Korea; 110 Department of Surgery, Seoul National University College of Medicine, Seoul, Korea; 111 Department of Genetics and Pathology, Pomeranian Medical University, Szczecin, Poland; 112 Postgraduate School of Molecular Medicine, Warsaw Medical University, Warsaw, Poland; 113 Molecular Epidemiology Unit, National Cancer Institute, Bangkok, Thailand; 114 Genetic Epidemiology Group, International Agency for Research on Cancer, Lyon, France; 115 Institute of Biomedical Sciences, Academia Sinica, Taipei, Taiwan; 116 Taiwan Biobank, Taipei, Taiwan; 117 Department of Surgery, Tri-Service General Hospital, Taipei, Taiwan; 118 Cancer Center and Department of Surgery, Kaohsiung Medical University Chung-Ho Memorial Hospital, Kaohsiung, Taiwan; 119 The Breakthrough Breast Cancer Research Centre, The Institute of Cancer Research, London, United Kingdom; 120 Section of Epidemiology, Institute of Cancer Research, Sutton, Surrey, United Kingdom; 121 University of Wisconsin Carbone Cancer Center, Madison, Wisconsin, United States of America; 122 Cancer Prevention Program, Fred Hutchinson Cancer Research Center, Seattle, Washington, United States of America; 123 Department of Community & Family Medicine, Department of Pediatrics, Dartmouth Medical School, Dartmouth-Hitchcock Medical Center, One Medical Center Drive, Lebanon, New Hampshire, United States of America; 124 Division of Population Sciences, Moffitt Cancer Center & Research Institute, Tampa, Florida, United States of America; Tsan Yuk Hospital, Hospital Authority, China

## Abstract

The 6q25.1 locus was first identified via a genome-wide association study (GWAS) in Chinese women and marked by single nucleotide polymorphism (SNP) rs2046210, approximately 180 Kb upstream of *ESR1*. There have been conflicting reports about the association of this locus with breast cancer in Europeans, and a GWAS in Europeans identified a different SNP, tagged here by rs12662670. We examined the associations of both SNPs in up to 61,689 cases and 58,822 controls from forty-four studies collaborating in the Breast Cancer Association Consortium, of which four studies were of Asian and 39 of European descent. Logistic regression was used to estimate odds ratios (OR) and 95% confidence intervals (CI). Case-only analyses were used to compare SNP effects in Estrogen Receptor positive (ER+) versus negative (ER**−**) tumours. Models including both SNPs were fitted to investigate whether the SNP effects were independent. Both SNPs are significantly associated with breast cancer risk in both ethnic groups. Per-allele ORs are higher in Asian than in European studies [rs2046210: OR (A/G) = 1.36 (95% CI 1.26–1.48), p = 7.6×10^−14^ in Asians and 1.09 (95% CI 1.07–1.11), p = 6.8×10^−18^ in Europeans. rs12662670: OR (G/T) = 1.29 (95% CI 1.19–1.41), p = 1.2×10^−9^ in Asians and 1.12 (95% CI 1.08–1.17), p = 3.8×10^−9^ in Europeans]. SNP rs2046210 is associated with a significantly greater risk of ER− than ER+ tumours in Europeans [OR (ER−) = 1.20 (95% CI 1.15–1.25), p = 1.8×10^−17^ versus OR (ER+) = 1.07 (95% CI 1.04–1.1), p = 1.3×10^−7^, p_heterogeneity_ = 5.1×10^−6^]. In these Asian studies, by contrast, there is no clear evidence of a differential association by tumour receptor status. Each SNP is associated with risk after adjustment for the other SNP. These results suggest the presence of two variants at 6q25.1 each independently associated with breast cancer risk in Asians and in Europeans. Of these two, the one tagged by rs2046210 is associated with a greater risk of ER− tumours.

## Introduction

A genome-wide association study (GWAS) in Chinese women by Zheng et al. [1] identified a novel breast cancer susceptibility locus at 6q25.1. The most strongly associated single nucleotide polymorphism (SNP) was rs2046210, with an estimated Odds ratio (OR) [per-allele A/G] = 1.29 (95% confidence interval (CI) 1.21–1.37, p = 10^−15^). SNP rs2046210 did not show a clear association in GWAS carried out in women of European ancestry, and replication studies indicated its effect, if any, was weaker in Europeans [OR (per allele A/G) = 1.04 (95% CI 0.99–1.08), p = 0.09 in a combined analysis of European studies [2]]. More recent studies in European women suggested stronger associations with other SNPs in the region: Turnbull et al. [3] found the most significantly associated SNP to be rs3757318, which is only weakly correlated with rs2046210 in Europeans (r^2^ = 0.09 from in HapMap2 CEU), while Stacey et al. [2] suggested that SNPs closer to *ESR1* may be more strongly associated. It is as yet unclear whether this difference in breast cancer associated SNPs between Asians and Europeans indicates the presence of a single or multiple causative variant(s) at this locus. If there is only one, it is unlikely to be highly correlated with the best tags identified from either the Asian or European GWAS and could potentially be a common variant with a small effect or a rarer one with a larger effect on breast cancer risk.

In this, by far the largest study to date, we investigate associations with SNP rs2046210, as well as with SNP rs12662670 in forty-four case-control studies within the Breast Cancer Association Consortium (BCAC). These two SNPs have been genotyped in a total of 120,511 female subjects, of which 110,265 subjects are of European ancestry and 8,559 are Asian. SNP rs2046210 is the best tag from the original Asian GWAS [1] and SNP rs12662670 is an easier to genotype surrogate for SNP rs3757318 - the best tag SNP at the 6q25.1 locus from a European GWAS [3]. Our aims were to compare the effects of these tags in well-powered studies of both Asian and European ancestry and to test if these known SNP associations are shared by the different ethnic groups. We have been successful in achieving these aims and our analyses provide additional insights into the nature of this locus.

## Materials and Methods

### Ethics Statement

Approval of the studies was obtained from the ethics committees listed in Table S1. All studies conform to the Declaration of Helsinki and all study participants gave written informed consent.

### Study Populations

Data from forty-five BCAC case-control studies from Australia, Europe, North America, and South-East Asia were available for inclusion in this analysis (see **Table S1** for a description of the individual studies). To be eligible for BCAC, studies needed to include at least 500 cases of invasive breast cancer and 500 controls, with DNA samples available for genotyping. The controls needed to be broadly from the same population as the cases (http://www.srl.cam.ac.uk/consortia/bcac/about/about.html). Some studies selected cases preferentially on the basis of age and/or family history.

All studies provided information on disease status (58,822 controls/62,061 invasive cases/2,769 *in-situ* cases/1,435 cases of unknown invasiveness), age at diagnosis or interview and ethnicity (Asian/European/other). Forty studies also provided information on estrogen receptor (ER) status for a total of 40,508 cases (9,878 Estrogen receptor negative (ER−)/30,630 Estrogen Receptor positive (ER+)).

### Laboratory Methods

In most studies SNPs were assayed by Taqman™ (Applied Biosystems, Foster City, USA). Primers, probes and master mix were ordered in a single batch and alliquots shipped to each study. Reactions were performed according to manufacturer’s instructions, using the following thermal cycling profile 95°C for 10 mins followed by: [92°C for 15 secs, 60°C for 1 min] for 40–60 cycles.

SNP rs12662670 was chosen as the most easily assayable surrogate for the best European GWAS hit, rs3757318, for which no working Taqman™ assay could be designed. These two SNPs are correlated at r^2^ = 0.89 in the European samples used in Turnbull et al. [3] although the correlations in populations of Asian ancestry are somewhat weaker (r^2^ = 0.72 and r^2^ = 0.66 in HapMap2 JPT and CHB samples, respectively).

The primer and probe sequences used were:

For **SNP rs2046210**

Forward primerTGCCTCAACTGTCTTGTGAATCTTT

Reverse primerCTACTGTAGAATCATTTTCCTCACACATACA

G allele probeVIC ACAGTCACATACGCATCTA

A allele probe FAM CAGTCACATACACATCTA

For **SNP rs12662670**

Forward primerCTAACGAAGGCAGAGCAAAAAGAAA

Reverse primerCACACATGCATGACACGTAAATCTT

T allele probeVIC ATTAAATTCTTGTAAGTTTCC

G allele probe FAM AATTCTTGTCAGTTTCC

Four studies (ACP, GESBC, kConFab/AOCS and MARIE) used the Sequenom iPLEX MassARRAY™ system (Sequenom, San Diego, CA, USA) with oligonucleotide design performed using MassARRAY Assay Design software (version 3.1).

SNPs were genotyped in three different BCAC genotyping phases along with other SNPs of interest to the consortium (see **Tables S2a and S2b** for information on the respective phases for SNPs rs2046210 and rs12662670). All studies followed standard quality control guidelines (for details see http://www.srl.cam.ac.uk/consortia/bcac/about/about.html). Data were excluded for any sample that failed genotyping for >20% of the SNPs typed in a given phase of genotyping. All study data were excluded for any SNP with overall call rate <95% or duplicate concordance <94% (based on at least 2% of samples in each study being genotyped in duplicate) or departure of genotype distribution from Hardy-Weinberg equilibrium in controls (p<0.005). In addition, all genotyping centres assayed an identical plate of 80 control DNA samples (referred to as the Coriell plate; which also included 14 internal duplicates) and had to achieve call rates and duplicate concordance >98% in order for their data to be included. Data for both SNPs from one study (NBCS) were excluded from further analyses after quality control rules were applied. Quality control data for the individual studies are shown in **Tables S2a and S2b**. Thus, for SNP rs2046210 forty out of forty-one assayed studies (56,607 cases/49,559 controls), and for SNP rs12662670 thirty-three out of thirty-four assayed studies (47,251 cases/40,161 controls) were included in the statistical analysis.

### Statistical Analyses

ORs were estimated using logistic regression. In order to provide reliable estimates of effect sizes, study-specific effect estimates of ORs were derived only for those studies that provided at least 100 cases and controls for the respective (sub-) group of interest.

The primary analysis estimated ORs for the main effect of the SNP, adjusted for the studies that provided data for the respective analysis (i.e. S-1 indicator variables were entered the logistic regression model, where S was number of studies that provided data for the respective analysis). ORs adjusted for both study and age were essentially identical and we did not therefore present the age-adjusted analyses. Per allele ORs were estimated under the assumption of a log-additive mode of inheritance, i.e. the SNP was coded according to the number of minor alleles 0, 1 or 2. Additionally, ORs by genotype were calculated, i.e. two indicator variables indicating the presence of the heterozygous genotype and the genotype homozygous for the minor allele, respectively, were entered the model. The primary p-values were derived by means of a Wald-Test assuming a log-additive mode of inheritance (one degree of freedom). Following Laird and Mosteller, heterogeneity of per allele ORs between studies was assessed by the p-value derived from the Q statistic [4] and using I^2^. Tests were two-sided.

Genetic main effects by ER status were estimated using case-control logistic regression and restricting the case sample to ER+ or ER− cases, respectively. To test for significant differences between main effects of rs2046210 or rs12662670 in ER+ versus ER− cases, logistic regression analyses were conducted in cases only. In these case-only analyses, the binary ER status was the outcome/dependent variable and the respective SNP and the indicator variables representing the studies were the independent variables.

Variation in OR by age was evaluated by testing for an interaction between age-group (<40, 40 to 49, 50 to 59, ≥60) and SNP, separately for each subgroup defined by ethnicity and ER status. Thus, the multiplicative SNP by age-group interaction term entered the model in addition to the main effect terms for SNP, age-group and study.

To investigate whether the association with breast cancer risk could be explained by one SNP or whether both SNPs had independent effects on disease risk, we fitted logistic regression models which included both SNPs, in addition to indicator variables for the studies, as independent variables in the model. Analyses were carried out separately for Europeans and Asians and for ER− versus ER+ cases and controls. Additionally, haplotype analyses were performed using logistic regression models that included the estimated two-marker haplotypes (coded according to a log-additive model) except for the reference haplotype (i.e., the most frequent haplotype) and the indicator variables for study. Haplotypes were estimated using the expectation-maximization algorithm.

All analyses, were performed using R version 2.11.0 [5] and the R packages meta, rmeta and haplo.stats.

## Results

**Figure 1:**
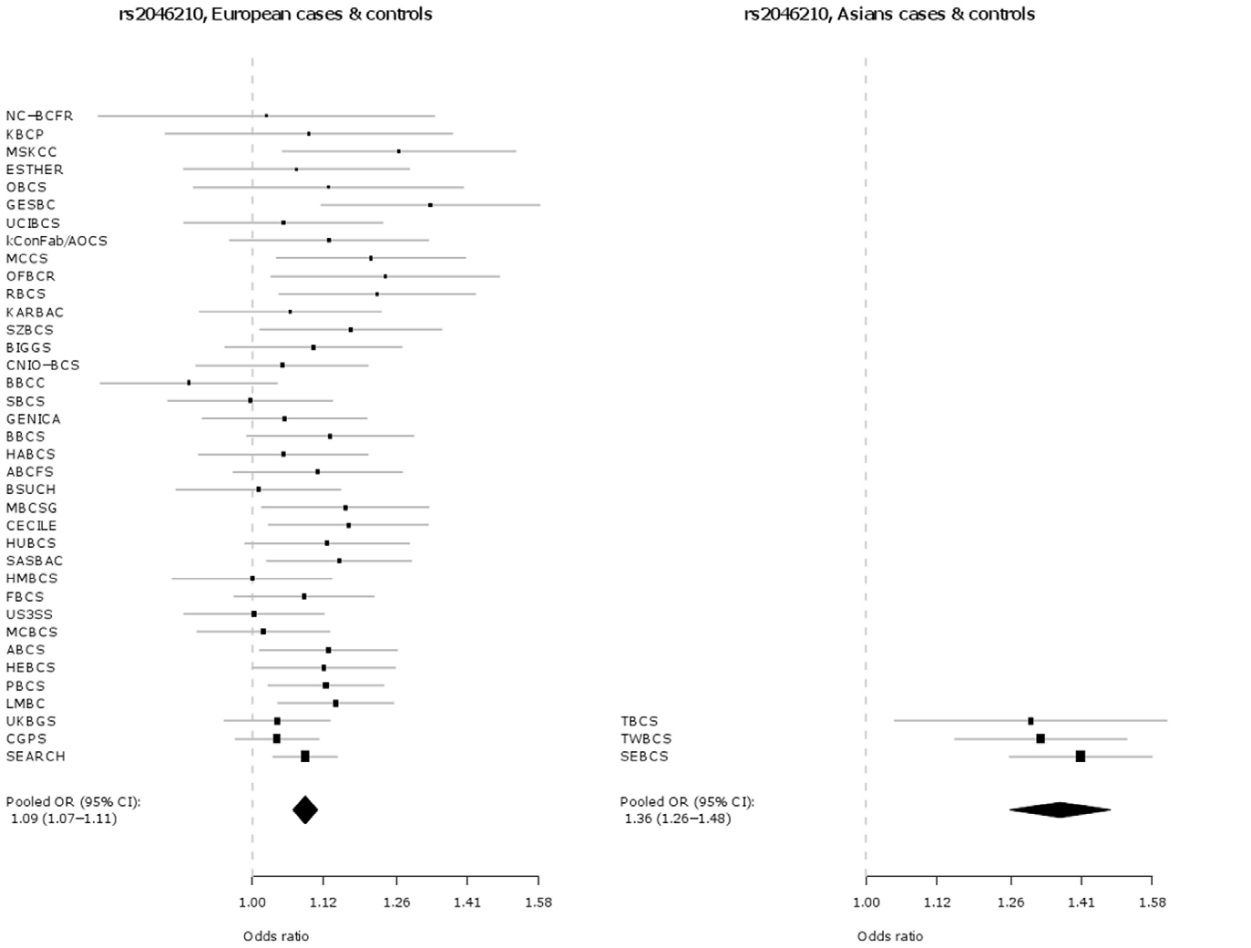
Association of rs2046210 with breast cancer in Europeans versus Asians.

**Figure 2:**
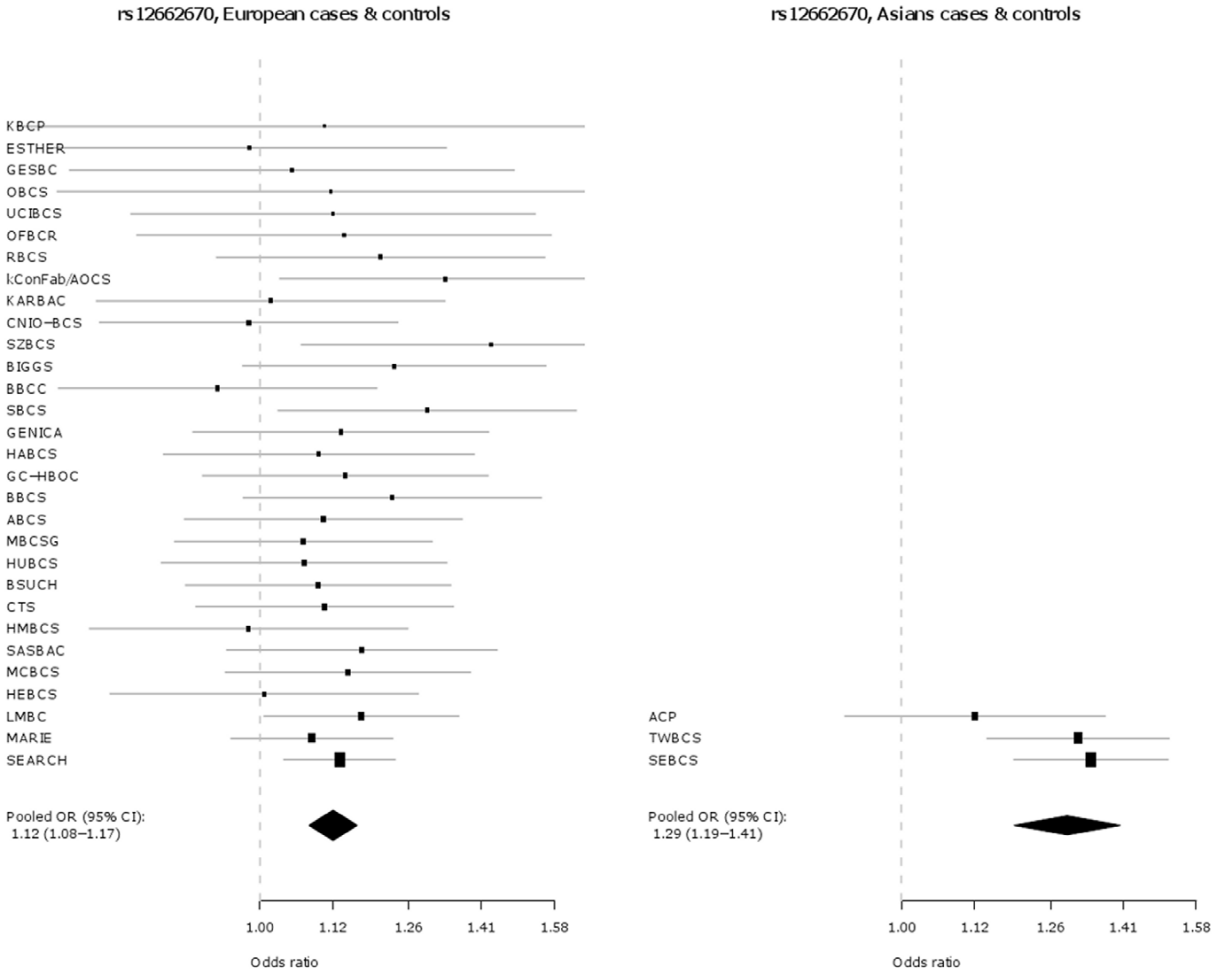
Association of rs12662670 with breast cancer in Europeans versus Asians.

Key characteristics for each participating study are shown in **Table S1**. In addition to the originally discovered SNP rs2046210, SNP s12662670 was genotyped as a surrogate for the best tag from Turnbull et al. (rs3757318) [3], for which no working Taqman™ assay could be designed. The genotype distributions by ethnicity and study for SNPs rs2046210 and rs12662670 in cases and controls are given in **Tables S3a and S3b**. The associations of each SNP are presented in **Table 1** and as Forest plots in **Figure 1** and **2**, separately for Europeans and Asians. Both SNPs are significantly associated with breast cancer risk in both ethnic groups. However, the per-allele OR associated with the minor A allele of SNP rs2046210 is higher in Asian populations [OR (A/G) = 1.36 (95% CI 1.26–1.48), p = 7.6×10^−14^] than in Europeans [OR (A/G) = 1.09 (95% CI 1.07–1.11), p = 6.8×10^−18^] and this difference is statistically significant [p_heterogeneity_  = 1.4×10^−7^]. SNP rs12662670 shows a similar pattern, with a higher OR associated with the minor G allele in Asian studies [OR (G/T) = 1.29 (95% CI 1.19–1.41), p = 1.2×10^−9^] than in Europeans [OR (G/T) = 1.12 (95% CI 1.08–1.17), p = 3.8×10^−9^] and again this difference is statistically significant [p_heterogeneity_  = 0.002]. In each case there is no evidence for departure from a log-additive model (a co-dominant mode of inheritance).

**Table 1:** Association of rs2046210 and rs12662670 with breast cancer.

Ethnicity	Number of cases/controls	Odds ratio (95% confidence interval)			P-value^a^
		per allele^b^	Heterozygote^c^	Homozygote^c^	
**rs2046210**					
Analyses adjusted for study					
*Overall*	54,647/49,559	1.10 (1.08–1.13)	1.11 (1.08–1.14)	1.22 (1.17–1.27)	3.30×10^−25^
*Europeans*	49,634/46,679	1.09 (1.07–1.11)	1.09 (1.06–1.12)	1.18 (1.13–1.23)	6.76×10^−18^
*Asians*	2983/2332	1.36 (1.26–1.48)	1.39 (1.23–1.56)	1.83 (1.54–2.18)	7.60×10^−14^
Analyses adjusted for study and rs12662570					
*Overall*	40,384/33,750	1.08 (1.05–1.11)	1.08 (1.05–1.12)	1.16 (1.10–1.23)	3.06×10^−9^
*Europeans*	36,396/31,105	1.08 (1.05–1.11)	1.08 (1.04–1.12)	1.16 (1.09–1.22)	1.78×10^−8^
*Asians*	3416/2420	1.17 (1.02–1.36)	1.18 (0.99–1.41)	1.37 (1.02–1.85)	0.028
**rs12662670**					
Analyses adjusted for study					
*Overall*	42,654/40,166	1.15 (1.12–1.19)	1.15 (1.10–1.19)	1.40 (1.23–1.58)	2.52×10^−16^
*Europeans*	38,723/37,400	1.12 (1.08–1.17)	1.13 (1.09–1.18)	1.12 (0.94–1.34)	3.83×10^−9^
*Asians*	3273/2451	1.29 (1.19–1.41)	1.24 (1.10–1.39)	1.77 (1.46–2.14)	1.18×10^−9^
Analyses adjusted for study and rs2046210					
*Overall*	40,384/33,750	1.10 (1.06–1.15)	1.10 (1.05–1.15)	1.25 (1.08–1.45)	5.86×10^−6^
*Europeans*	36,396/31,105	1.07 (1.02–1.12)	1.09 (1.03–1.14)	1.01 (0.83–1.23)	2.91×10^−3^
*Asians*	3416/2420	1.21 (1.04–1.40)	1.16 (0.97–1.38)	1.55 (1.12–2.13)	0.012

Results are presented overall and separately for Europeans and Asians. Pooled analyses adjusted for study only as well as adjusted for rs12662670 or rs2046210, respectively, in addition to study were performed.

a P-value derived from the log-additive model.

b Odds ratio per minor allele (A allele for rs2046210, G allele for rs12662670).

c Odds ratio relative to the major allele homozygous (GT) genotype.

Logistic regression models, which include both SNPs, indicate that the two SNPs are independently associated in both Europeans (p = 1.8×10^−8^ for rs2046210, p = 2.9×10^−3^ for rs12662670; **Table 1**) and Asians (p* = *0.028 for rs2046210, p = 0.012 for rs12662670). In each ethnicity the estimated ORs for each SNP, after adjustment for the other SNP, are of similar magnitudes: For rs2046210 in Europeans OR (A/G) = 1.08 (95% CI 1.05–1.11) and for rs12662670 in Europeans OR (G/T) = 1.07 (95% CI 1.02–1.12). For rs2046210 in Asians OR (A/G) = 1.17 (95% CI 1.02–1.36) and for rs12662670 in Asians OR (G/T) = 1.21 (95% CI 1.04–1.40). Similar effect estimates are also obtained for haplotypes carrying one minor allele though estimates do not reach statistical significance for the very rare haplotype carrying the major (G) allele of rs2046210 along with the minor (G) allele of rs12662670 (**Table 2**). Of note, from the four observed haplotypes, effects are strongest and highly statistically significant for the haplotype carrying both minor alleles: In Europeans OR (AG) = 1.16 (95% CI 1.11–1.21). In Asians OR (AG) = 1.42 (95% CI 1.30–1.56).

**Table 2:** Association of haplotypes composed of rs2046210 and rs12662670 with breast cancer.

Ethnicity	Number of cases/controls	rs2046210	rs12662670	Haplotype frequency	Odds ratio (95% confidence interval)^a^	P-value^b^
*Overall*	40,384/33,750	A^c^	T	0.26	1.08 (1.05–1.11)	1.55×10^−9^
		G	G^c^	0.01	1.10 (0.95–1.27)	0.189
		A^c^	G^c^	0.09	1.23 (1.18–1.28)	2.20×10^−4^
		G^d^	T^d^	0.64^d^	–	–
*Europeans^e^*	36,396/31,105	A^c^	T	0.27	1.08 (1.05–1.11)	1.59×10^−8^
		G	G^c^	0.01	1.02 (0.86–1.21)	0.832
		A^c^	G^c^	0.07	1.16 (1.11–1.21)	4.35×10^−11^
		G^d^	T^d^	0.65^d^	–	–
*Asians^e^*	3416/2420	A^c^	T	0.09	1.23 (1.05–1.45)	1.15×10^−2^
		G	G^c^	0.04	1.23 (0.94–1.61)	0.134
		A^c^	G^c^	0.27	1.42 (1.30–1.56)	4.44×10^−14^
		G^d^	T^d^	0.60^d^	–	–

Results are presented overall and separately for Europeans and Asians. Pooled analyses adjusted for study were performed.

a Odds ratio per haplotype compared to the reference haplotype (i.e., the most frequent haplotype).

b P-value derived from the log-additive model.

c Minor allele.

d Reference haplotype.

The OR estimates for *in-situ* cancer are similar to those for invasive cancer for both SNPs in Europeans, although, due to small numbers, the effect of rs12662670 on *in-situ* tumours does not reach statistical significance (**Table S4**). For each Asian study, the number of *in-situ* cases is less than 100 and so effect estimates are inaccurate but do not differ from those for invasive cancer (data not shown).

**Figure 3:**
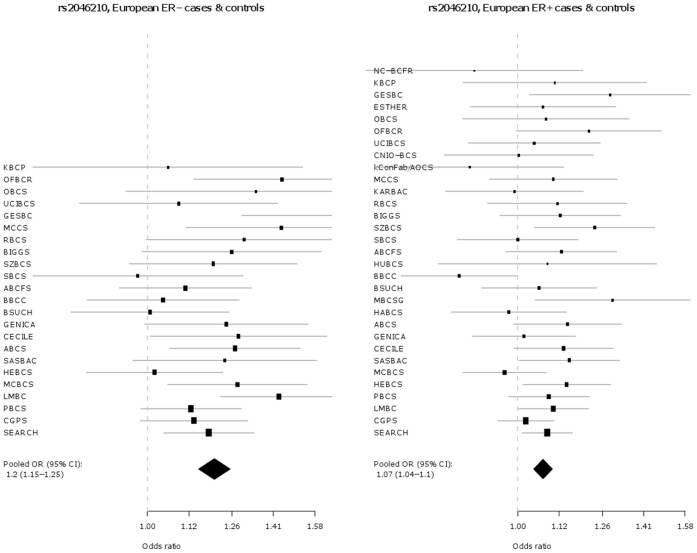
Association of rs2046210 with breast cancer in European ER −*versus ER+*cases and controls. *Estrogen receptor negative; **Estrogen receptor positive.

**Figure 4:**
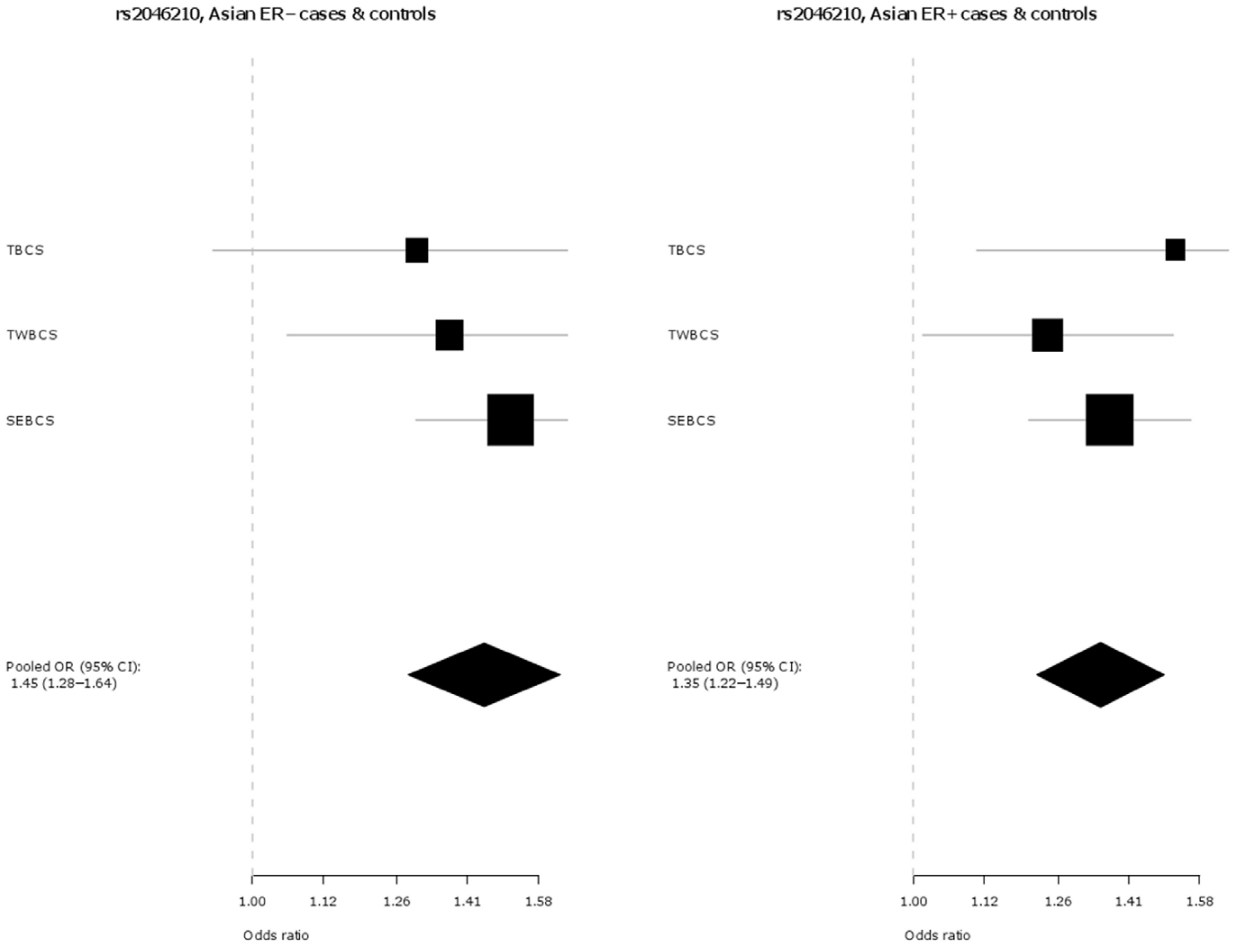
Association of rs2046210 with breast cancer in Asian ER −*versus ER+**cases and controls. *Estrogen receptor negative; **Estrogen receptor positive.

**Figure 5:**
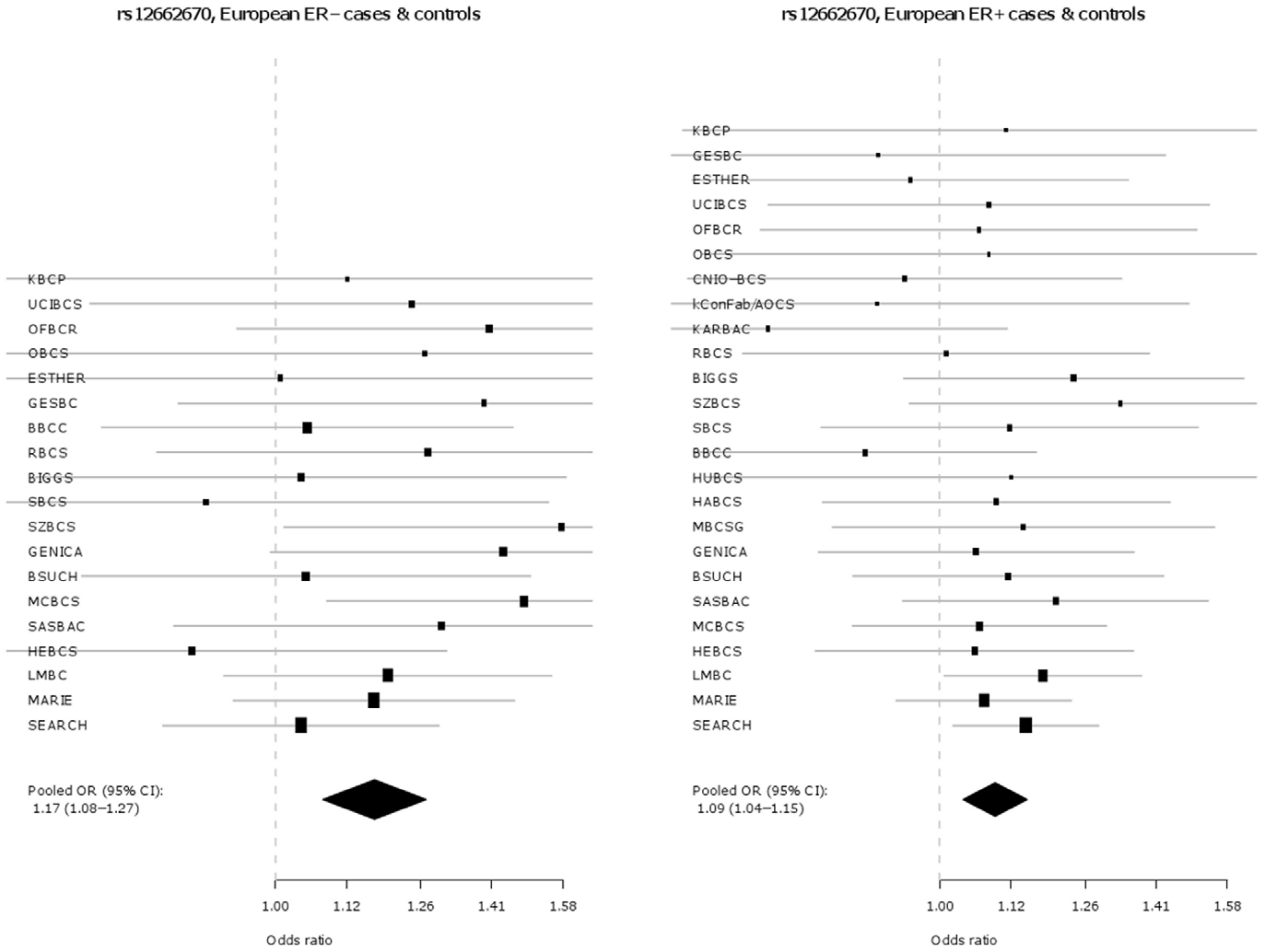
Association of rs12662670 with breast cancer in European ER −*versus ER+**cases and controls. *Estrogen receptor negative; **Estrogen receptor positive.

**Figure 6:**
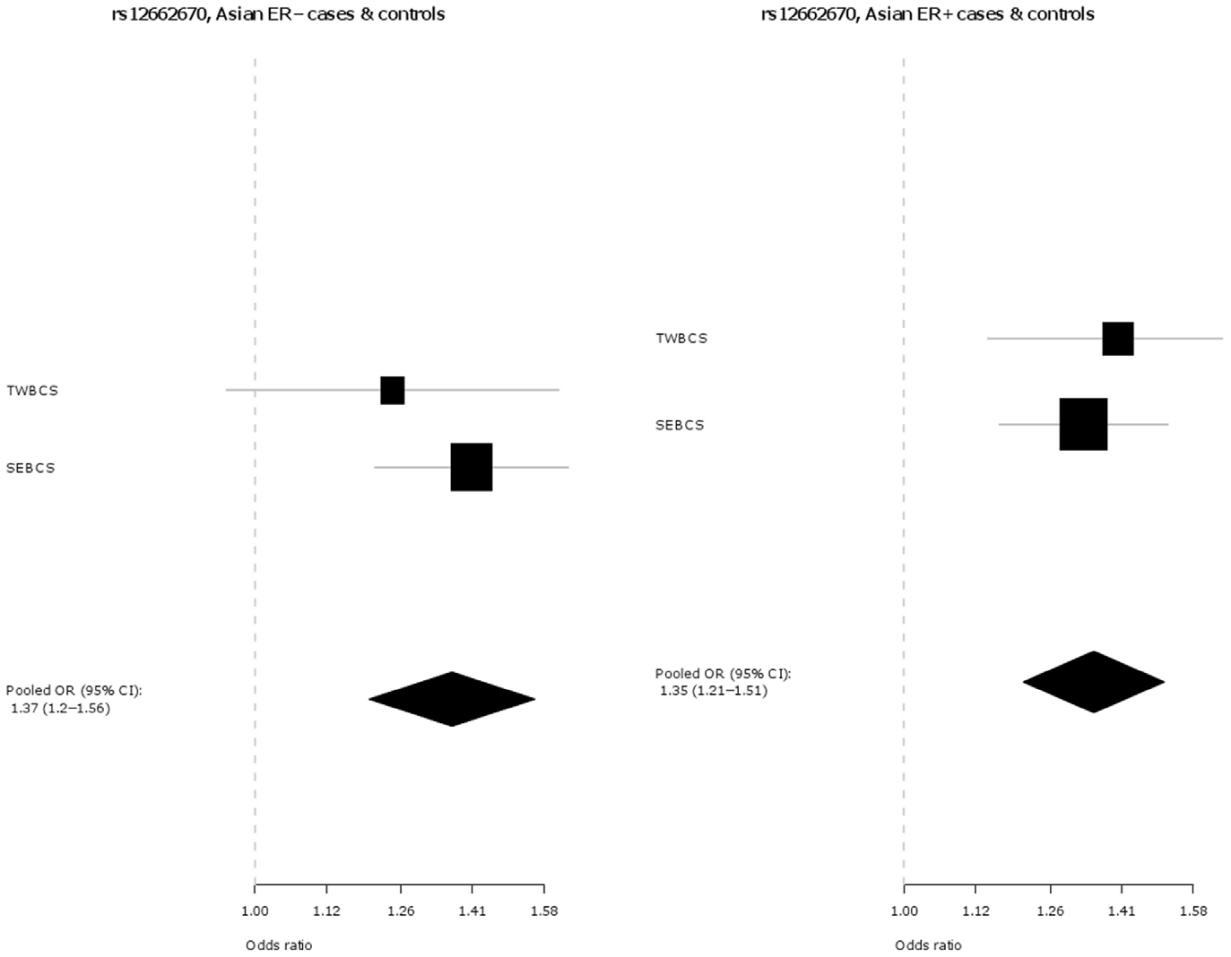
Association of rs12662670 with breast cancer in Asian ER −*versus ER+*cases and controls. *Estrogen receptor negative; **Estrogen receptor positive.

The associations of these two SNPs with tumour sub-types defined by ER status (ER+ and ER−) were also investigated and are presented in **Table 3** and **Figures 3**, **4**, **5**, and **6**. In Europeans, SNP rs2046210 is associated with a greater OR for ER− than ER+ tumours: OR (ER−) = 1.20 (95% CI 1.15–1.25), p = 1.8×10^−17^ vs. OR (ER+) = 1.07 (95% CI 1.04–1.1), p = 1.3×10^−7^, p_heterogeneity_  = 5.1×10^−6^. This difference remains significant after adjustment for rs12662670. A similar, although non-significant, difference is observed in European women for SNP rs12662670 (**Table 3**). In the Asian studies, however, there is no clear evidence of a differential association by tumour receptor status for either SNP (**Table 3**).

**Table 3:** Association of rs2046210 and rs12662670 with risk of ER−*/ER+** breast cancer.

Ethnicity	Estrogen receptor status	Number of cases/controls	Odds ratio (95% confidence interval)^a^	P-value^b^	P-heterogeneity^c^
**rs2046210**					
Analyses adjusted for study					
*Overall*	*ER*−*	7126/35,833	1.23 (1.18–1.28)	6.90×10^−25^	
	*ER+* **	5914/32,977	1.09 (1.06–1.12)	1.66×10^−11^	5.93×10^−7^
*Europeans*	*ER*−*	807/2334	1.20 (1.15–1.25)	1.77×10^−17^	
	*ER+* **	24,596/42,664	1.07 (1.04–1.10)	1.26×10^−7^	5.10×10^−6^
*Asians*	*ER*−*	22,336/39,856	1.45 (1.29–1.64)	1.70×10^−9^	
	*ER+* **	1307/2334	1.35 (1.22–1.49)	1.09×10^−8^	0.513
Analyses adjusted for study and rs12662670					
*Overall*	*ER*−*	5526/25,627	1.19 (1.13–1.26)	1.26×10^−10^	
	*ER+* **	19,502/32,010	1.07 (1.03–1.10)	7.26×10^−5^	1.01×10^−3^
*Europeans*	*ER*−*	4497/23,090	1.20 (1.13–1.26)	4.54×10^−10^	
	*ER+* **	17,653/29,365	1.07 (1.03–1.10)	2.16×10^−4^	4.63×10^−4^
*Asians*	*ER*−*	975/2420	1.16 (0.94–1.44)	0.169	
	*ER+* **	1649/2420	1.13 (0.94–1.35)	0.194	0.777
**rs12662670**					
Analyses adjusted for study					
*Overall*	*ER*−*	5422/28,201	1.23 (1.15–1.32)	6.16×10^−9^	
	*ER+* **	4563/26,198	1.14 (1.09–1.19)	3.05×10^−8^	0.074
*Europeans*	*ER*−*	808/1890	1.17 (1.08–1.27)	1.90×10^−4^	
	*ER+* **	20,095/34,460	1.09 (1.04–1.15)	6.08×10^−4^	0.070
*Asians*	*ER*−*	18,519/32,349	1.37 (1.20–1.56)	3.30×10^−6^	
	*ER+* **	1394/1890	1.35 (1.21–1.51)	1.58×10^−7^	0.691
Analyses adjusted for study and rs2046210					
*Overall*	*ER*−*	5526/25,627	1.10 (1.01–1.20)	0.028	
	*ER+* **	19,502/32,010	1.09 (1.03–1.15)	2.14×10^−3^	0.87
*Europeans*	*ER*−*	4497/23,090	1.03 (0.94–1.14)	0.516	
	*ER+* **	17,653/29,365	1.05 (0.99–1.11)	0.110	0.92
*Asians*	*ER*−*	975/2420	1.33 (1.07–1.65)	0.011	
	*ER+* **	1649/2420	1.26 (1.04–1.52)	0.017	0.30

Results are presented overall as well as separately for Europeans and Asians. Pooled analyses adjusted for the studies were performed. A log-additive genetic model was assumed.

* Estrogen receptor negative.

** Estrogen receptor positive.

a Odds ratio per minor allele (A allele for rs2046210, G allele for rs12662670) derived from case-control logistic regression restricted to ER+ or ER− cases, respectively, and the whole control sample.

b P-value derived from the log-additive model derived from case-control logistic regression restricted to ER+ or ER− cases, respectively, and the whole control sample.

c P-value for heterogeneity between estimates of genetic main effects derived from case-only analysis.

We further investigated whether the magnitudes of these SNP associations on tumour sub-types differed by age at diagnosis/interview (see **Table S5**). In Asian studies the data are too sparse to give meaningful results. In the combined ethnicities and the European studies alone, the magnitudes of the observed associations are greater in younger women.

Fourteen of the European studies had been designed to over-sample cases with a family history of breast cancer (see **Table S1**), which could have led to an overestimation of the ORs relative to those expected in a population-based case-control study. However, exclusion of these studies does not materially affect the estimated ORs for either SNP (see **Table S6**).

## Discussion

In this large collaborative study of up to 61,689 cases and 58,822 controls, we demonstrate a highly statistically significant association between the A allele of rs2046210 and increased breast cancer risk in women of both Asian and European ancestry, thus extending the association previously observed in Asian populations. Consistent with previous reports [1]–[3], the effect sizes are significantly greater in Asians than in Europeans. Our study also reveals that the G allele of SNP rs12662670 is significantly associated with increased breast cancer risk in both ethnicities. SNP rs12662670 is used here as surrogate for SNP rs3757318 - the most strongly associated SNP at this locus in the European GWAS described by Turnbull et al. [3]. In addition, and also in contrast to Stacey et al. [2], we find that the OR for rs12662670 is greater in Asians than in Europeans (**Table 1**, **Figure 1** and **2**). In contrast to previous reports, our study indicates that both SNPs (rs2046210 and rs12662670) may be independently associated with breast cancer risk – in models including both SNPs, both maintain significant ORs after adjustment for the other. Haplotype analyses result in effect estimates for the AT and GG haplotypes, which carry only one minor allele, very similar to those of the single SNP analyses for the respective minor alleles. Furthermore, haplotype analyses show a clearly stronger effect of the AG haplotype, carrying both minor alleles, compared to the effects of the AT and GG haplotypes, further supporting the hypothesis that there may be two different causative variants, one on each haplotype carrying only one minor allele and both on the haplotype carrying both minor alleles (i.e., the stronger effect of the AG haplotype compared to the AT and GG haplotypes may be explained by the joint effect of the two minor alleles on the AG haplotype). However, the alternative conclusion that a single causative variant may exist that is intermediate between the two SNPs phylogenetically, i.e. on the AG haplotype and on some of the AT haplotypes, cannot yet be completely excluded, since this could also be an explanation for the stronger effect of the AG haplotype compared to the AT and GG haplotypes.

We also find evidence that SNP rs2046210 is more strongly associated with ER− than ER+ disease in both European and Asian women. In the present study this differential association with receptor status is statistically significant in European studies (and remains after adjustment for rs12662670) but is not quite significant in Asians which may be due to a lack of power attributable to the comparatively small number of Asian individuals involved in our study (**Table 3**). However this same SNP had previously been reported to be more strongly associated with ER− tumours in the original Chinese cases [1] as well as in a recent replication study in Chinese women [6]. In line with these reports, a meta-analysis (14,231 cases, 10,244 controls) on this SNP-disease association by ER status in Asians, incorporating published results as well as those presented here, reveals a significant difference in OR associated with ER− versus ER+ tumour risk [OR (A/G - ER− ) = 1.37 (95% CI 1.30–1.44), p = 3.7×10^−33^ vs. OR (A/G- ER+) = 1.27 (95% CI 1.22–1.34), p = 2.2×10^−24^; p_heterogeneity_  = 0.04]. A stronger association of SNP rs2046210 with ER− tumours is also consistent with the report from the Consortium of Modifiers of BRCA1/2 (CIMBA) [7] that the same allele is associated with an increased Hazard Ratio of breast cancer in BRCA1 mutation carriers (who predominantly develop ER− tumours). The CIMBA study also observed that this allele conferred increased Hazard Ratios among younger mutation carriers while we observed similar trends for greater SNP ORs at younger age groups (**Table S5**). By contrast, the CIMBA consortium reported that SNP rs9397435 (the tag they used for rs12662670; r^2^ = 0.61, r^2^ = 0.50 and r^2^ = 0.85 in HapMap2 CEU, JPT and CHB samples, respectively) shows evidence of modification of risk in both BRCA1 and BRCA2 mutation carriers (who mainly develop ER− and ER+ tumours respectively) [7] whilst similarly, we find that SNP rs12662670 is associated with increased risks of both ER− and ER+ tumours.

Previous fine-scale mapping publications on this locus [2], [8] have sought a single variant to explain the associations seen with all SNPs in the region: Stacey et al. [2] proposed SNP rs9397435 as a possible single causative variant since it was more strongly associated than rs2046210 in women of European, African and Asian ancestry. We are not able to comment on this variant, as it has not been genotyped in BCAC. However, our findings suggest there could be two independent associations at this locus: one, better tagged by SNP rs2046210, predisposing to ER− tumours and the second, better tagged by rs12662670, conferring similar risks of both tumour types. Although physically close, SNPs rs2046210 and rs12662670 are not highly correlated with each other, particularly in Europeans (in BCAC r^2^ = 0.12 in Europeans and r^2^ = 0.56 in Asians) and all four possible combinations (haplotypes) of these two SNPs clearly exist.

**Figure 7:**
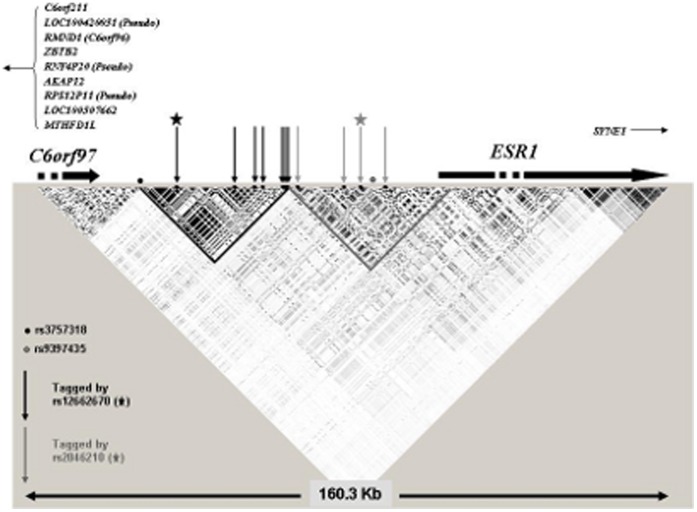
Linkage disequilibrium blocks in the *ESR1* region. Five SNPs tagged (at r2>0.9) by rs12662670 and three by rs2046210 are marked by arrows (dark and light grey respectively); rs12662670 and rs2046210 are marked by stars; rs3757318 and rs9397435 are marked by points; blocks were generated using data from the 1000 Genomes Project and HapMap; blocks include all single nucleotide polymorphisms with a minor allele frequency >0.05. The directions of translation of *ESR1* and *C6orf97* are marked and other genes in the locus are listed.

Examination of linkage disequilibrium plots of the regions surrounding these two SNPs in Europeans (**Figure 7**) reveals little, if any, physical overlap between SNPs highly correlated (r^2^>0.9) with rs2046210 and those with rs12662670. If there were a single causal variant, directly responsible for the associations seen with both SNPs, it would need to be correlated with both SNPs. Such a variant has not been yet identified (e.g. by the 1000 Genomes Project). It would presumably be relatively rare. An alternative, and we think, more plausible, explanation for the pattern of associations may be the existence of two independent causative variants, one correlated with rs2046210 and another correlated with rs12662670. If this is the case, the former variant may be more strongly associated with ER− breast cancer than the latter. The reason why both SNPs confer higher relative risks in Asians than in Europeans is unclear. Within the BCAC studies, ER− tumours are relatively more prevalent among Asian (36%) compared to European cases (23%), but this is not sufficient to explain the higher ORs in Asians, since the effects persist after stratification by ER status. It remains possible that the higher relative risks are due to differential patterns of linkage disequilibrium if the, as yet, unidentified causal variants are not strongly correlated with the SNPs identified to date. These questions may be resolved by comprehensive re-sequencing of this locus and fine scale mapping to identify the causal variant (or variants) responsible for the observed breast cancer risks. One aim of the iCOGS Project [9], which is currently underway, is to address these questions. However it is possible that these observed differences between Asians and Europeans may reflect interactions with lifestyle risk factors or other unlinked genetic loci. Another possible explanation is that the estimated SNP effects in Asians are inflated given the phenomenon known as the “winner’s curse”, i.e. the suboptimal power of the pool of Asian studies (due to the small number of Asian individuals) together with the commonly used requirement for a published association to pass a certain pre-defined p-value threshold may have resulted in biased SNP effect estimates [10], [11].

Although there are eleven genes within 1 Mb of this locus, attention has focused on the *ESR1* gene, whose transcription start site is located approximately 180 Kb downstream of SNP rs2046210. *ESR1* encodes ERα and has long been implicated in breast carcinogenesis. However, it is possible that the proximity of this SNP to *ESR1* may be providing a false lead – both SNPs (rs2046210 and rs12662670) lie in the flanking region of *C6orf97* and there are numerous other genes in close physical proximity (see **Figure 7**). It is notable however, that SNPs mapping to this region have also been identified in GWAS for bone mineral density – another phenotype in which estradiol metabolism is clearly implicated [12], [13]. Furthermore, a recent paper [14] demonstrates that a number of genes, including *ESR1* and *C6orf97* are co-regulated at this locus although the functions of most of these co-regulated genes have not yet been elucidated. The SNP associations, presented here, may provide a basis to explore the biological role of this locus in estrogen signalling and cancer development in more detail.

Taken together our findings suggest the possibility of the presence of two different causative variants at the 6q25.1 locus and indicate that fine-scale mapping efforts aimed at finding a single variant accounting for associations with both marker SNPs, may not be successful.

## Supporting Information

Table S1 **Characteristics of 45 case-control studies within the Breast Cancer Association Consortium (BCAC).**
(DOC)

Table S2 **Characteristics of the study populations genotyped for rs2046210 (a) and rs12662670 (b).**
(DOC)

Table S3 **Genotype frequencies of SNP rs2046210 (a) and rs12662670 (b) in the different studies.**
(DOC)

Table S4 **Association of rs2046210 and rs12662670 with in-situ/invasive breast cancer.**
(DOC)

Table S5 **Association of rs2046210 and rs12662670 with risk of ER**−*/ER+**breast cancer.
(DOC)

Table S6 **Association of rs2046210 and rs12662670 with breast cancer.**
(DOC)

## References

[1] Zheng W, Long J, Gao YT, Li C, Zheng Y, et al (2009) Genome-wide association study identifies a new breast cancer susceptibility locus at 6q25.1. *Nat Genet* 41: 324–328. 19219042 10.1038/ng.318 PMC2754845

[2] Stacey SN, Sulem P, Zanon C, Gudjonsson SA, Thorleifsson G, et al (2010) Ancestry-shift refinement mapping of the C6orf97-ESR1 breast cancer susceptibility locus. *PLoS Genet* 6: e1001029. 20661439 10.1371/journal.pgen.1001029 PMC2908678

[3] Turnbull C, Ahmed S, Morrison J, Pernet D, Renwick A, et al (2010) Genome-wide association study identifies five new breast cancer susceptibility loci. *Nat Genet* 42: 504–507. 20453838 10.1038/ng.586 PMC3632836

[4] Laird NM, Mosteller F (1990) Some statistical methods for combining experimental results. *Int J Technol Assess Health Care* 6: 5–30. 2361819 10.1017/s0266462300008916

[5] R Development Core Team (2005) R: A language and environment for statistical computing. Vienna, Austria, R Foundation for Statistical Computing.

[6] Long J, Shu XO, Cai Q, Gao YT, Zheng Y, et al (2010) Evaluation of breast cancer susceptibility Loci in chinese women. *Cancer Epidemiol Biomarkers Prev* 19: 2357–2365. 20699374 10.1158/1055-9965.EPI-10-0054 PMC2936687

[7] Antoniou AC, Kartsonaki C, Sinilnikova OM, Soucy P, McGuffog L, et al (2011) Common alleles at 6q25.1 and 1p11.2 are associated with breast cancer risk for BRCA1 and BRCA2 mutation carriers. *Hum Mol Genet* 20(16): 3304–3321. 21593217 10.1093/hmg/ddr226 PMC3652640

[8] Cai Q, Wen W, Qu S, Li G, Egan KM, et al (2011) Replication and Functional Genomic Analyses of the Breast Cancer Susceptibility Locus at 6q25.1 Generalize Its Importance in Women of Chinese, Japanese, and European Ancestry. *Cancer Res* 71: 1344–1355. 21303983 10.1158/0008-5472.CAN-10-2733 PMC3083305

[9] The Collaborative Oncological Gene-Environment Study (COGS) Project. Available: http://www.cogseu.org. Accessed 2012 Jul 10.

[10] Ioannidis JPA (2008) Why most discovered true associations are inflated. Epidemiology 19(5): 640–648. 18633328 10.1097/EDE.0b013e31818131e7

[11] Kraft P (2008) Curses – winner’s and otherwise – in genetic epidemiology. Epidemiology 19(5): 649–651. 18703928 10.1097/EDE.0b013e318181b865

[12] Li WF, Hou SX, Yu B, Li MM, Ferec C, et al (2010) Genetics of osteoporosis: accelerating pace in gene identification and validation. *Hum Genet* 127: 249–285. 20101412 10.1007/s00439-009-0773-z

[13] Xie H, Sun M, Liao XB, Yuan LQ, Sheng ZF, et al (2010) Estrogen receptor-alpha36 mediates a bone-sparing effect of 17beta-estrodiol in postmenopausal women. *J Bone Miner Res* 26: 156–168. 10.1002/jbmr.169 PMC3179309 20578216

[14] Dunbier AK, Anderson H, Ghazoui Z, Lopez-Knowles E, Pancholi S, et al (2011) ESR1 Is Co-Expressed with Closely Adjacent Uncharacterised Genes Spanning a Breast Cancer Susceptibility Locus at 6q25.1. *PLoS Genet* 7: e1001382. 21552322 10.1371/journal.pgen.1001382 PMC3084198

