# Correction: Comparison of 6q25 Breast Cancer Hits from Asian and European Genome Wide Association Studies in the Breast Cancer Association Consortium (BCAC)

**DOI:** 10.1371/annotation/e5de602c-0ffc-4e6f-a2ed-f79913c2e57c

**Published:** 2012-10-18

**Authors:** Rebecca Hein, Melanie Maranian, John L. Hopper, Miroslaw K. Kapuscinski, Melissa C. Southey, Daniel J. Park, Marjanka K. Schmidt, Annegien Broeks, Frans B. L. Hogervorst, H. Bas Bueno-de-Mesquit, Kenneth R. Muir, Artitaya Lophatananon, Suthee Rattanamongkongul, Puttisak Puttawibul, Peter A. Fasching, Alexander Hein, Arif B. Ekici, Matthias W. Beckmann, Olivia Fletcher, Nichola Johnson, Isabel dos Santos Silva, Julian Peto, Elinor Sawyer, Ian Tomlinson, Michael Kerin, Nicola Miller, Frederick Marmee, Andreas Schneeweiss, Christof Sohn, Barbara Burwinkel, Pascal Guénel, Emilie Cordina-Duverger, Florence Menegaux, Thérèse Truong, Stig E. Bojesen, Børge G. Nordestgaard, Henrik Flyger, Roger L. Milne, Jose Ignacio Arias Perez, M. Pilar Zamora, Javier Benítez, Hoda Anton-Culver, Argyrios Ziogas, Leslie Bernstein, Christina A. Clarke, Hermann Brenner, Heiko Müller, Volker Arndt, Christa Stegmaier, Nazneen Rahman, Sheila Seal, Clare Turnbull, Anthony Renwick, Alfons Meindl, Sarah Schott, Claus R. Bartram, Rita K. Schmutzler, Hiltrud Brauch, Ute Hamann, Yon-Dschun Ko, The GENICA Network, Shan Wang-Gohrke, Thilo Dörk, Peter Schürmann, Johann H. Karstens, Peter Hillemanns, Heli Nevanlinna, Tuomas Heikkinen, Kristiina Aittomäki, Carl Blomqvist, Natalia V. Bogdanova, Iosif V. Zalutsky, Natalia N. Antonenkova, Marina Bermisheva, Darya Prokovieva, Albina Farahtdinova, Elza Khusnutdinova, Annika Lindblom, Sara Margolin, Arto Mannermaa, Vesa Kataja, Veli-Matti Kosma, Jaana Hartikainen, Xiaoqing Chen, Jonathan Beesley, kConFab Investigators, AOCS Group, Diether Lambrechts, Hui Zhao, Patrick Neven, Hans Wildiers, Stefan Nickels, Dieter Flesch-Janys, Paolo Radice, Paolo Peterlongo, Siranoush Manoukian, Monica Barile, Fergus J. Couch, Janet E. Olson, Xianshu Wang, Zachary Fredericksen, Graham G. Giles, Laura Baglietto, Catriona A. McLean, Gianluca Severi, Kenneth Offit, Mark Robson, Mia M. Gaudet, Joseph Vijai, Grethe Grenaker Alnæs, Vessela Kristensen, Anne-Lise Børresen-Dale, Esther M. John, Alexander Miron, Robert Winqvist, Katri Pylkäs, Arja Jukkola-Vuorinen, Mervi Grip, Irene L. Andrulis, Julia A. Knight, Gord Glendon, Anna Marie Mulligan, Jonine D. Figueroa, Montserrat García-Closas, Jolanta Lissowska, Mark E. Sherman, Maartje Hooning, John W. M. Martens, Caroline Seynaeve, Margriet Collée, Per Hall, Keith Humpreys, Kamila Czene, Jianjun Liu, Angela Cox, Ian W. Brock, Simon S. Cross, Malcolm W. R. Reed, Shahana Ahmed, Maya Ghoussaini, Paul DP. Pharoah, Daehee Kang, Keun-Young Yoo, Dong-Young Noh, Anna Jakubowska, Katarzyna Jaworska, Katarzyna Durda, Elżbieta Złowocka, Suleeporn Sangrajrang, Valerie Gaborieau, Paul Brennan, James McKay, Chen-Yang Shen, Jyh-Cherng Yu, Huan-Ming Hsu, Ming-Feng Hou, Nick Orr, Minouk Schoemaker, Alan Ashworth, Anthony Swerdlow, Amy Trentham-Dietz, Polly A. Newcomb, Linda Titus, Kathleen M. Egan, Georgia Chenevix-Trench, Antonis C. Antoniou, Manjeet K. Humphreys, Jonathan Morrison, Jenny Chang-Claude, Douglas F. Easton, Alison M. Dunning

The tenth author's name was spelled incorrectly. The correct name is: H. Bas Bueno-de-Mesquita.

