# Supplementary material for: Comparison of 6q25 Breast Cancer Hits from Asian and European Genome Wide Association Studies in the Breast Cancer Association Consortium (BCAC)
Source: PLoS One. 2012 Aug 7;7(8):e42380. doi: 10.1371/journal.pone.0042380 (PMC3413660; doi:10.1371/journal.pone.0042380)
Supplement: Table S1 — Characteristics of 45 case-control studies within the Breast Cancer Association Consortium (BCAC). (DOC) [file pone.0042380.s001.doc]

**Table S1: Characteristics of 45 case-control studies within the Breast Cancer Association Consortium (BCAC).**

| **Study** | **Abbreviation** | **Case Definition and Ascertainment** | **Control Definition and Ascertainment** | **Participation Rates** | **Number of Cases / Controls** | **Age Range for Cases / Controls** | **Approval committee** |
| --- | --- | --- | --- | --- | --- | --- | --- |
| Australian Breast Cancer Family Study [1] | ABCFS | All cases diagnosed < age 40 plus a random sample of those diagnosed ages 40-59 from cancer registries in Victoria and New South Wales, plus a limited number diagnosed aged 60-69; cases living in Melbourne recruited from 1992-99 and in Sydney from 1993-98. | Identified from the electoral rolls in Melbourne from 1992-98 and Sydney from 1993-99. Frequency matched to cases by age in 5 year categories. | 75% of cases and 68% of controls completed questionnaires, 71% of cases and 55% of controls provided a blood sample | 1423 / 755 | 23-69 / 20-68 | The University of Melbourne Health Sciences Human Ethics Sub-Committee (HESC) |
| Amsterdam Breast Cancer Study [2] | ABCS  ABCS-F | All cases (operable, invasive mammacarcinoma) aged <50 and diagnosed from 1974-1994 in 4 Dutch hospitals (in Amsterdam and Leiden).  All non-BRCA1/2 breast cancer cases from the family cancer clinic of the NKI-AVL tested in the period 1995-2009; all ages and diagnosed with breast cancer in 1965-2008. | Random selection of women <50 years of age at baseline from 2 population-based prospective studies - the Monitoring Project on Cardiovascular Risk Factors (1987-1991) and the Monitoring Project on Chronic Disease Risk Factors (1993-1997). These studies were run by National Institute for Public Health and the Environment, The Netherlands. Controls were from the same catchment area as cases. | ~85% of cases and ~50% of controls for DNA (blood/paraffin). | 2555 / 974 | 23-79 / 20-49 | Leiden University Medical Center (LUMC) Commissie Medische Ethiek and Protocol Toetsingscommissie van het Nederlands Kanker Instituut/Antoni van Leeuwenhoek Ziekenhuis |
| Asian Cancer Program | ACP | 1. Women who underwent biopsy and have been pathologically diagnosed as having breast cancer.  2. Women younger than 71 years of age | 1. Women younger than 71 years without cancer history of any kinds.  2. Women who attend the outpatient clinic under the minor injuries such as cuts, broken bones.  3. Women who are institutionalised at the hospital with diseases not related to cancer or metabolic syndromes such as diabetes, heart diseases or conditions related to gynaecology and are well enough to give information to researchers. | Cases and controls were invited to join the study and 100% of consented subjects were interviewed and provided information to the study. Over 95% of both case and control group provided blood samples. | 324 / 561 | 21-70 / 18-70 | Ethics Committee of National Cancer Institute Thailand; Prince of Songkla University Faculty of Medicine Ethics Committee; Khon Kaen University Ethics Committee for Human Research; HRH Princess Maha Chakri Sirindhorn Medical Centre (MSMC) Ethics Committee |
| Bavarian Breast Cancer Cases and Controls [3] | BBCC | Consecutive, unselected cases with invasive breast cancer recruited at the University Breast Centre, Franconia in Northern Bavaria during 2002-2006. | Healthy women with no diagnosis of cancer aged 55 or older. Invited by a newspaper advertisement in Northern Bavaria, and recruited during 2002-2006 | 95% of cases and 99% of controls provided a blood sample and an epidemiological questionnaire. | 1315 / 899 | 22-96 / 18-100 | Friedrich-Alexander-Universitat Erlangen-Nurnberg Medizinische Fakultat Ethik-Commission |
| British Breast Cancer Study [4] | BBCS | 1) English & Scottish Cancer Registries: all breast cancer cases who developed a first primary before age 65 in 1971 or later and who subsequently developed a second primary cancer. 2) Breast cancer clinics: all breast cancer cases who developed a first primary before age 71 in 1967 or later and who either subsequently developed a second primary or had at least two affected female first degree relatives. | 1) A friend, sister-in-law, daughter-in-law or other non-blood relative of cases. Recruitment of cases and controls was from January 2001 to the end of 2008. | 1) 68% of cases & 76% of controls provided a blood sample. | 1150 / 827 | 26-77 / 21-81 | South East Multi-Centre Research Ethics Committee |
| Breast Cancer in Galway Genetic Study [5] | BIGGS | Unselected cases recruited from West of Ireland since 2001. Cases were recruited from University College Hospital Galway and surrounding hospitals | Women > 60 years with no personal history of any cancer and no family History of breast or ovarian cancer were identified from retirement groups in the West of Ireland (same catchment area as cases) during the period 2001-2008. | Not recorded | 920 / 838 | 24-90 / 25-96 | Galway University College Hospital Clinical Research Ethical Committee |
| Breast Cancer Study of the University Clinic Heidelberg [6,7] | BSUCH | All cases diagnosed with breast cancer in 2007-2009 at the University Women`s Clinic Heidelberg | Healthy, unrelated, ethnically matched female blood donors recruited in 2007 & 2009 by German Red Cross Blood Service of Baden-Württemberg-Hessen, Institute of Transfusion Medicine & Immunology, Mannheim. | Cases: 82% Controls:Offers of blood donation from approximately 5% of donors were refused due to various reasons | 1101 / 1314 | 25-89 / 30-69 | Medizinische Fakultat Heidelberg Ethikkommission |
| CECILE Breast Cancer Study [8] | CECILE | All cases diagnosed with breast cancer in 2005-2007 among women <75 years of age and residing in Ille-et-Vilaine (Rennes) or in Côte d'Or (Dijon) at diagnosis. Cases were recruited from the main cancer treatment center (Centre Eugène-Marquis in Rennes and Centre Georges-François-Leclerc in Dijon) and from private or public hospitals in each area. | General population control women residing in the same areas. Controls were recruited in 2005-2007 using a random digit dialing procedure and quotas by socioeconomic status, and were frequency-matched to the cases by 5-year age groups. | Face to face interview completed for 77% of cases, and 74% of controls. Among interviewed subjects, 85% of cases and 78% of controls provided a blood sample, and 12 % of cases and 19% of controls provided DNA from buccal cell samples | 1067 / 1037 | 25-74 / 25-74 | Comite Consultatif de Protection des Personnes dans la Recherche Biomedicale de Bicetre |
| Copenhagen General Population Study [9] | CGPS | Consecutive, incident cases from 1 hospital with centralized care for a population of 400,000 women from 2001 to the present. | Community controls residing in the same region as cases and with no history of breast cancer were identified from the Copenhagen General Population Study recruited 2003-2007. All controls were known to still be breast cancer-free at the end of 2007. | 96% of cases and 56% of controls were interviewed and provided a blood sample. | 2722 / 6548 | 24-95 / 20-91 | Kobenhavns Amt den Videnskabsetiske Komite |
| Spanish National Cancer Centre Breast Cancer Study [10] | CNIO-BCS | Two groups of cases:1) 574 consecutive breast cancer patients, unselected for family history, from 3 public hospitals, 2 in Madrid and one in Oviedo, from 2000 to 2005. 2) 291 cases with at least one first degree relative also affected with breast cancer, recruited through the CNIO family cancer clinic in Madrid from 2000 to 2004. | Women attending the Menopause Research Centre between 2000 and 2004 and female members of the College of Lawyers attending a free, targeted medical check-up in 2005, all free of breast cancer and all in Madrid | Not recorded. | 953 / 804 | 23-88 / 26-86 | Hospital Universitario La Paz Comite Etico de Investigacion Clinica |
| California Teachers Study | CTS | This is a nested case-control study conducted within a cohort of California teachers(113,590) who were under age 80 years at baseline, had no prior history of  invasive or *in situ* breast cancer. Cases are  women newly diagnosed with a histologically confirmed invasive primary adenocarcinoma of the breast at age 80 years or younger from 1998 to 2008. | Controls are a probability sample of at-risk cohort members, frequency matched to cases on age at baseline (5-year age  groups), self-reported race/ethnicity (white, African American, Latina,Asian, other), and broad geographic region within California  Controls were selected without replacement, using an assigned reference  date. |  | 1372 / 1315 | 32-83 / 26-77 | UC Irvine: Office of Research Institutional Review Board |
| ESTHER Breast Cancer Study [11] | ESTHER | Statewide recruitment of breast cancer cases in all hospitals in Saarland/Germany in 2001-2003 (ESTHER) and 1996-1998 (VERDI) | Statewide recruitment of participants of a routine health check-up in Saarland/Germany in 2000-2002. A stratified random sample, matched to the cases by five year age groups, was selected as controls. | Not recorded | 500 / 507 | 30-79 / 49-75 | Ruprecht-Karls-Universitat Medizinische Fakultat Heidelberg Ethikkommission |
| ICR Familial Breast Cancer Study [12] | FBCS | Index patients with breast cancer and at least 2 relatives affected with breast cancer. All White individuals collected in 1995-2005 throughout UK | White individuals collected throughout UK from 1958 Birth Cohort Collection, an ongoing follow-up of persons born in UK in one week in 1958 | not applicable | 1728 / 1043 | 18-87 / - | The London Multi-Centre Research Ethics Committees |
| German Consortium for Hereditary Breast & Ovarian Cancer [13] | GC-HBOC | Index patients from German breast cancer families; BRCA1/2 mutation free, collected 1996-2007 via Institute of Human Genetics, University Heidelberg & Department of Gynaecology & Obstetrics, Cologne & Department of Gynaecology and Obstetrics at the Ludwig-Maximilians-University, Munich; Germany. | Healthy, unrelated, ethnically matched female blood donors recruited in 2004 & 2007 by German Red Cross Blood Service of Baden-Württemberg-Hessen, Institute of Transfusion Medicine & Immunology, Mannheim. | Cases: About 3% refused to spend their blood samples for research purposes. Controls: Offers of blood donation from approximately 5% of donors were refused due to various reasons | 847 / 1124 | 19-87 / 19-68 | Ethik-Kommission der Medizinischen Fakultat der Universitat zu Koln |
| Gene Environment Interaction and Breast Cancer in Germany [14,15] | GENICA | Incident breast cancer cases enrolled between 2000 and 2004 from the Greater Bonn area (by of the hospitals within the study region); all enrolled within 6 months of diagnosis | Selected from population registries from 31 communities in the greater Bonn area; matched to cases in 5-year age classes between 2001 and 2004 | Response rate 88% for cases and 67% for controls. Of these, DNA samples are available for 89% and 90% respectively. | 959 / 980 | 23-80 / 24-80 | Rheinische Friedrich-Wilhelms-Universitat Medizinische Einrichtungen Ethik-Kommission |
| Genetic Epidemiology Study of Breast Cancer by Age 50 [16] | GESBC | All incident cases diagnosed <50 years of age in 1992-5 in two regions: Rhein-Neckar-Odenwald and Freiburg, by surveying the 38 clinics serving these regions | Selected from random lists of residents of the study regions supplied by population registries; two controls were selected for each case, matched by age and study region. Recruitment was carried out 1992-1998. | 70.2% of cases and 61.2% of controls completed the questionnaire. | 553 / 556 | 24-50 / 24-52 | Ruprecht-Karls-Universitat Medizinische Fakultat Heidelberg Ethikkommission |
| Hannover Breast Cancer Study [17] | HABCS | Cases who received radiotherapy for breast cancer at Hannover Medical School between 1997-2003, unselected for age or family history | Anonymous female blood bank donors at Hannover Medical School, collected from 8/2005-12/2005, with known age and ethnic background | Approx. 80% of cases and 70% of controls contacted agreed to give a blood sample | 1029 / 997 | 25-91 / 18-68 | Medizinische Hochschule Hannover Ethik-Kommission |
| Helsinki Breast Cancer Study [18-20] | HEBCS | (1) Consecutive cases (883) from the Department of Oncology, Helsinki University Central Hospital 1997-8 and 2000, (2) Consecutive cases (986) from the Department of Surgery, Helsinki University Central Hospital 2001 – 2004, (3) Familial breast cancer patients (536) from the Helsinki University Central Hospital, Departments of Oncology and Clinical Genetics (1995-) | Healthy females from the same geographical region in Southern Finland in 2003. | ( 1) 79% of all cases for the 1. consecutive series, (2) 87% of all cases for the 2. consecutive series 87% , (3) about 90% of the familial cases. Controls (100%). | 2370 / 1255 | 22-96 / 18-66 | Helsingin ja uudenmaan sairaanhoitopiiri (Helsinki University Central Hospital Ethics Committee) |
| Hannover-Minsk Breast Cancer Study [21] | HMBCS | Ascertainment at the Byelorussian Institute for Oncology and Medical Radiology Aleksandrov N.N. in Minsk or at one of 5 regional oncology centers in Gomel, Mogilev, Grodno, Brest or Vitebsk through the years 2002-2008. | Controls from the same population aged 18-72 years. Healthy (without personally history of cancer) female probunds recruited from the same geographical regions as cases during the years 2002-2008. About 75% of controls were women invited for general medical examination at five regional gynecology clinics (in Gomel, Mogilev, Grodno, Brest or Vitebsk ) and cancer-free volunteers ascertained at the Institute for Inherited Diseases in Minsk; 20% were cancer-free female blood bank donors recruited at Republic Blood Bank, Minsk, Belarus; finally 5% of controls were healthy cancer-free relatives of some breast cancer patients. | More than 60% for cases and more than 80% for controls. DNA available from all of the participants. | 1656 / 913 | 16-82 / 18-72 | Medizinische Hochschule Hannover Ethik-Kommission |
| Hannover-Ufa Breast Cancer Study [21] | HUBCS | Consecutive Russian breast cancer patients aged 24-86 years ascertained at one of the two participating oncological centers in Bashkorstostan and Siberia through the years 2000-2008 | Population controls aged 18-84 years recruited from a population study of different populations of Russia. Healthy volunteers (without any malignancy) were selected from the same geographical regions during the years 2002-2008. | Not assessed | 943 / 1414 | 25-85 / - | Ethical Committee of Institute of Biochemistry and Genetics Ufa Science Center |
| Karolinska Breast Cancer Study [22,23] | KARBAC | 1. Familial cases from Department of Clinical Genetics, Karolinska University Hospital , Stockholm. 2. Consecutive cases from Department of Oncology, Huddinge & Söder Hospital, Stockholm 1998-2000 | Blood donors of mixed gender from same geographical region. Excess material was received from all blood donors over a 3 month period in 2004 (approximately 3000) and DNA was extracted from a random sample of 1500 | 1. NA 2. 70% of consecutive cases provided a blood-sample | 810 / 843 | 24-88 / - | Lokala Forskningsetikkommitten Nord |
| Kuopio Breast Cancer Project [24] | KBCP | Women seen at Kuopio University Hospital between 1990 and 1995 because of breast lump, mammographic abnormality, or other breast symptom who were found to have breast cancer | Age and long-term area-of-residence matched controls selected from the National Population Register and interviewed in parallel with the cases | Cases: 98% of those contacted; which is 86% of those potentially eligible. Controls were selected individually for each case (response rate not available) | 488 / 397 | 23-92 / 27-77 | Pohjois-Savon Sairraanhoitopiirin Kuntayhtyma Tutkimuseettinen Toimikunta |
| Kathleen Cuningham Foundation Consortium for research into Familial Breast Cancer/Australian Ovarian Cancer Study [25] | KConFab/AOCS | Cases were from multiple-case breast and breast-ovarian families recruited though family cancer clinics from across Australia and New Zealand from 1998 to the present. Cases were selected for inclusion in BCAC studies if (i) family was negative for mutations in BRCA1 and BRCA2 (ii) case was the index for the family, defined as youngest breast cancer affected family member. | Female controls were ascertained by the Australian Ovarian Cancer Study identified from the electoral rolls from all over Australia from 2002-2006. | 78% family members approached agreed to participate. Of those, 97% provided a blood sample and 96% provided questionnaire data | 726 / 979 | 17-78 / 20-83 | kConFab: The Queenland Institute of Medical Research Human Research Ethics Committee (QIMR-HREC); AOCS: Peter MacCallum Cancer Centre Ethics Committee |
| Leuven Multidisciplinary Breast Centre [26] | LMBC | All patients diagnosed with breast cancer and seen in the Multidisciplinary Breast Center in Leuven (Gashuisberg) since June 2007 plus retrospective collection of cases diagnosed since 2000 | Healthy controls (blood donors) collected at the Red Cross and located in Gasthuisberg hospital (Oct-2007-March 2008) | High participation rate. At least 90% of patients and controls participated to studies. Few people are unwilling. | 2950 / 1603 | 22-94 / 19-66 | Commissie Medische Ethiek van de Universitaire Ziekenhuizen Kuleuven |
| Mammary Carcinoma Risk Factor Investigation [27] | MARIE | Incident and prevalent cases diagnosed from 2001-2005 in the study region Hamburg in Northern Germany, and from 2002-2005 in the study region Rhein-Neckar-Karlsruhe in Southern Germany. | 2 controls per case were randomly drawn from population registries and frequency matched by birth year and study region to the case. Controls were recruited from 2002 to 2006. | 64.1% of cases & 43.4% of controls participated (rates for people with QX) | 2546 / 4904 | 50-75 / 49-75 | Ruprecht-Karls-Universitat Medizinische Fakultat Heidelberg Ethikkommission |
| Milan Breast Cancer Study Group [28] | MBCSG | Familial and/or early onset breast cancer patients (aged 22-87) negative for mutations in BRCA genes, ascertained in two large cancer centres in Milan from May 1996 (centre 1) and May 2000 (centre 2) to July 2008 | Healthy blood donors aged 18-71 years, recruited at two blood centres in Milan from February 2004 to March 2009 (centre 1) and from November 2007 to January 2009 (centre 2). | Not applicable | 748 / 1346 | 21-80 / 18-71 | Comitato Etico Indipendente della Fondazione IRCCS "Istituto Nazionale dei Tumori" |
| Mayo Clinic Breast Cancer Study [29] | MCBCS | Incident cases residing in 6 states (MN, WI, IA, IL, ND, SD) seen at the Mayo Clinic in Rochester, MN from 2002-5 | Women without cancer presenting for general medical examination at the Mayo Clinic. Controls were recruited concurrently with cases and were frequency matched to cases on age, ethnicity and county/state | 68% for cases, 77% for controls were interviewed and provided a blood sample | 1839 / 1567 | 22-93 / 25-86 | Mayo Clinic IRB |
| Melbourne Collaborative Cohort Study [30] | MCCS | Incident cases diagnosed within the Melbourne Collaborative Cohort Study during the follow-up from baseline (1990-1994) to 2008 of the 24469 participating women | Random sample of the initial cohort | All incident cases and all the controls in the random sample. DNA available from >95% of the participants. | 668 / 758 | 37-80 / 38.4-70.2 | The Cancer Council Victoria Human Research Ethics Committee |
| Memorial Sloan-Kettering Cancer Center [31] | MSKCC | Incident and prevalent breast cancer patients who were referred to the Clinical Genetics Service at MSKCC. All tested negative for BRCA1/2 mutations. | Unaffected patients who were referred to the Clinical Genetics Service at MSKCC. All tested negative for BRCA1/2 mutations. | Not recorded | 535 / 480 | 21-85* / 24-86.3 | Memorial Sloan-Kettering Cancer Center IRB |
| Norwegian Breast Cancer Study [32] | NBCS | Incidence cases from three different hospitals: 1) Cases (114) mean age 64 (28-92) at Ullevål Univ. Hospital 1990-94, 2) cases (182) mean age 59 (26-75) referred to Norwegian Radium Hospital 1975-1986, 3) cases (124), mean age 56 (29-82) ) with stage I or II disease, in the Oslo micro-metastases study at Norwegian Radium Hospital between 1995-1998, 4) cases (71) mean age 67 (37–82) with locally advanced disease at Haukeland Univ. Hospital. | Control subjects were healthy women, age 55-71, residing in Tromsø (440), and Bergen (109) attending the Norwegian Breast Cancer Screening Program. | 80-82% cases and 70% controls | 1678 / 1891 | 26-90 / 22-75 | Regional Komite for Medisinsk Forskningsetikk (Helseregion III Universitetet I Bergen, Universitetet I Oslo, Helseregion Sor, Helseregion II, and Ost-Norge) |
| Northern California Breast Cancer Family Registry [33] | NC-BCFR | Cases included those enrolled in the NC-BCFR as part of Phase I and II recruitment. Incident cases aged <65 years diagnosed between 1995 and 2003 were identified through the SEER cancer registry of the Greater San Francisco Bay Area. All cases likely at increased genetic risk were eligible to enroll in the NC-BCFR (dx at age <35 yrs, personal history of ovarian or childhood cancer, bilateral breast cancer with 1st dx at age <50, family history of breast or ovarian cancer in first-degree relatives). Cases not meeting these criteria were randomly sampled (2.5% of whites, 30% of African Americans, 28% of Hispanics, 38% of Asian Americans). | Controls were identified through random digit dialing conducted from 1999-2000 in the same geographic region. Controls were frequency matched to cases diagnosed from 1995-1998 on 5-year age group and race/ethnicity, at a ratio of 1 control per 2 cases. | Cases identified by the cancer registry were screened by telephone to assess study eligibility. Of those contacted (alive, with MD permission and valid address), 90% completed the screening interview. Of cases eligible to enroll in the NC-BCFR, 77% completed the epidemiology and family history questionnaires, and 67% provided a blood or mouthwash sample. Of RDD controls, 60% completed the questionnaires, and 56% provided a biospecimen sample. | 1612 / 334 | 22-65 / 19-66 | Cancer Prevention Institute of California IRB |
| Oulu Breast Cancer Study | OBCS | Consecutive incident cases diagnosed at the Oulu University Hospital between 2000 and 2004. | Healthy, consecutive, anonymous, female Finnish Red-Cross blood donors recruited in 2002 from the same geographical region in Northern Finland. | All of the asked controls, and 71% of all cases treated at the Oulu University Hospital, Department of Oncology during the collection period. | 537 / 497 | 28-92 / 18-66 | Ethical Committee of the Medical Faculty of University of Oulu and Northern Ostrobothnia Hospital District Ethical Committee |
| Ontario Familial Breast Cancer Registry [33] | OFBCR | Cases diagnosed between 1 Jan 1996-31 Dec 1998 were identified from the Ontario Cancer Registry which registers >97% of all cases residing in the province at the time of diagnosis. All women with invasive breast cancer aged 20–54 years who met the OFBCR definition for high genetic risk (family history of specific cancers particularly breast and ovarian, early onset disease, Ashkenazi ethnicity or a diagnosis of multiple breast cancer) were asked to participate by completing risk factor questionnaires and providing a blood sample. A 25% random sample of individuals in this age category who did not meet the OFBCR definition, 35% of those aged 55–69 at high risk and 8.75% aged 55–69 at low risk were also asked to participate. This multi-step sampling scheme enriched the population for genetically predisposed individuals, which was an objective of the Ontario Familial Breast Cancer Registry. | Unrelated, unaffected population controls were recruited between 1998-2001 by calling randomly selected residential telephone numbers throughout the same geographical region. Eligible controls were women with no history of breast cancer and were frequency-matched by 5-year age group to the expected age distribution of cases. Approximately, 65% of identified eligible women returned questionnaires, and 63% of these donated a blood specimen. | Cases: consent to contact patients was 92%, response to initial family history questionnaire was 65%, response to risk factor questionnaires was 73% of all eligible, and donation of a blood sample was 63% of all eligible. Less than 2% died before initial contact. Controls: approximately, 65% of identified eligible women returned questionnaires, and 63% of these donated a blood specimen. | 1387 / 365 | 22-81 / 26-69 | Mount Sinai Hospital Research Ethics Board |
| NCI Polish Breast Cancer Study [34] | PBCS | Incident cases from 2000-2003 identified through a rapid identification system in participating hospitals covering ~ 90% of all eligible cases, and cancer registries in Warsaw and Łódź covering 100% of all eligible cases | Randomly selected from population lists of all residents of Poland, stratified and frequency matched to cases by case city and age in 5 year categories. Recruited 2000-2003. | 79% of eligible cases and 69% of eligible controls agreed to personal interview; 84% of interviewed cases and 94% of interviewed controls provided a DNA sample | 2083 / 2288 | 27-75 / 24-75 | National Institute of Health (NIH) IRB |
| Rotterdam Breast Cancer Study [35] | RBCS | Familial breast cancer patients selected from the clinical genetics center at Erasmus Medical Center; recruited 1994 - 2005 | Spouses or mutation-negative siblings of heterozygous Cystic Fibrosis mutation carriers selected from the clinical genetics center at Erasmus Medical Center; recruited 1996 - 2006 | 100% of cases and controls provided a blood sample. 95% of cases have signed the informed consent. All controls are anonymous and therefore no informed consent is nescessary. | 749 / 786 | 18-84 / - | Medische Ethische Toetsings Commissie Erasmus Medisch Centrum |
| Singapore and Sweden Breast Cancer Study [36] | SASBAC | Incident cases from October 1993 to March 1995 identified via the 6 regional cancer registries in Sweden, to which reporting is mandatory. | Controls were randomly selected from the total population registry in 5-year age groups to match the expected age-frequency distribution among cases. Patients and controls were recruited from Oct 1993 through April 1995. | 84% of cases & 82% of controls questionnaire, 87% & 74% of those donated DNA (overall 73% & 61% respectively). | 1234 / 1477 | 50-75 / 49-76 | Regionala Etikprovningsnamnden i Stockholm (Regional Ethical Review Board in Stockholm) |
| Sheffield Breast Cancer Study [37] | SBCS | Women with pathologically confirmed breast cancer recruited from surgical outpatient clinics at the Royal Hallamshire Hospital, Sheffield, 1998 – 2005; cases are a mixture of prevalent and incident disease | Unselected women attending the Sheffield Mammography Screening Service between Sep 2000 - Aug 2004, if their mammograms showed no evidence of a breast lesion | Not recorded | 975 / 953 | 28-92 / 45-78 | South Sheffield Research Ethics Committee |
| Study of Epidemiology and Risk factors in Cancer Heredity [38] | SEARCH | 2 groups of cases identified through East Anglian Cancer Registry; 1) prevalent cases diagnosed age <55 from 1991-6 and alive when study started in 1996; 2) incident cases diagnosed age < 70 diagnosed after 1996 | Two groups of controls: (1) selected from the EPIC-Norfolk cohort study of 25,000 individuals age 45-74 recruited between 1992 and1994, based in the same geographic region as cases; (2) selected from GP practices from March 2003 to present, frequency matched to cases by age and geographic region | 64% of eligible cases and 41% of invited controls provided a blood sample | 6553 / 6747 | 23-69 / 26-81 | Multi Centre Research Ethics Committee (MREC) |
| Seoul Breast Cancer Study [39,40] | SEBCS | Consecutive, incident, cases from 2 hospitals in Seoul recruited 2001-2005 | Healthy community controls from same catchment area and participating in annual health check-up, 2001-2005. | ~ 85% of cases, 75% of controls were interviewed and provided a blood sample. | 2522 / 1512 | 19-82 / 18-86 | Seoul National University College of Medicine/Seoul National University Hospital IRB |
| IHCC-Szczecin Breast Cancer Study [41] | SZBCS | Prospectively ascertained cases of invasive breast cancer patients diagnosed at the Regional Oncology Hospital (Szczecin) in the years 2002, 2003, 2006 and 2007 or the University Hospital from 2002 to 2007 in Szczecin, West-Pomerania, Poland. Patients with pure intraductal or intralobular cancer were excluded (DCIS or LCIS) but patients with DCIS with micro-invasion were included. | Unaffected, matched to cases for year of birth, sex and region; from families with negative cancer family history; controls were part of a population- based study of the 1.3 million inhabitants of West Pomerania performed in 2003 and 2004 designed to identify familial aggregations of cancer by our centre | >95% cases and 55% controls | 916 / 930 | 25-88 / 26-91 | Komisji Bioetycznej Pomorskiej Akademii Medycznej |
| IARC-Thai Breast Cancer Study [42] | TBCS | Incident cases diagnosed at the National Cancer Institute (NCI) in Bangkok and Khon Kaen Hospital during the period May 2002-March 2004. | Controls were randomly selected healthy females visiting hospital patients with diseases other than breast or ovarian cancer at NCI Bangkok and Khon Kaen Hospital during the period May 2002-March 2004. | 94% of cases and 73% of controls completed a questionnaire and provided a blood sample | 431 / 292 | 17-81 / 18-72 | IARC Institutional Review Board Committee |
| Taiwanese Breast Cancer Study [43,44] | TWBCS | Incident cases diagnosed & treated at 2 major teaching hospitals in Taiwan. [between March 2002 and August 2005] | Controls cancer-free individuals, randomly selected from women attending health exam. at same hospital during study period. Underwent 1-day health examination - any showing evidence cancer excluded. | >90% cases & ~ 40% of controls | 894 / 908 | 18-85 / 25-81 | Human Subject Research Ethics Committee/IRB Academia Sinica |
| UCI Breast Cancer Study [45] | UCIBCS | All cases diagnosed in Orange County, California, during one-year period beginning March 1, 1994. Ascertained through the population-based Cancer Surveillance Program of Orange County California (CSPOC) | Female controls under age 75 years without history of cancer recruited using random digit dialing among Orange County residents & frequency matched to cases by age & race/ethnicity. Recruited from 1998-2003 | Cases 76% and Controls 80% | 1052 / 562 | 24-90 / 20-75 | UC Irvine: Office of Research Institutional Review Board |
| UK Breakthrough Generations Study | UKBGS | All members who had had breast cancer before entry into the Breakthrough Generations Study (cohort of 100,000+ women followed up for breast cancer, recruited from the UK during 2003-2009). | Women who had not had breast cancer before entry into the cohort study, 1:1 matched to cases on date of birth, year of entry into the study (2003-2009), source of recruitment, blood sample and ethnicity | All selected subjects were recruited from within the cohort study | 2312 / 2366 | 24-84 / 26-86 | South East Multi-Centre Research Ethics Committee |
| US Three State Study [46] | US3SS | Eligible cases were all English-speaking female residents of Massachusetts (excluding metropolitan Boston), New Hampshire and Wisconsin, with a new diagnosis of invasive (aged 20–69 years) or in situ breast cancer (aged 20–74 years, MA and NH only) reported to each state's mandatory cancer registry during 1998-2001. | Controls were randomly selected from driver’s license lists for women aged 20–64 years and from Medicare beneficiary lists for women aged 65–74 years in each state, and frequency-matched to cases by age in 5-year categories. Recruited 1998-2001. | Approximately 80% of eligible breast cancer cases and 75% of eligible controls provided a telephone interview on known or suspected breast cancer risk factors. Buccal cell samples were collected by mail as a DNA source using cytobrush or mouthwash collection kits. Participation rates for buccal cell collection among cases and controls who completed the interview were 73 and 64%, respectively, for cytobrush samples, and 71 and 61%, respectively, for mouthwash samples. | 1635 / 1267 | 29-73 / 28-77 | National Institute of Health (NIH) IRB |

*Values provided are for age at interview in cases

**Reference List**

1. Dite GS, Jenkins MA, Southey MC, Hocking JS, Giles GG et al. (2003) **Familial risks, early-onset breast cancer, and BRCA1 and BRCA2 germline mutations.** *J Natl Cancer Inst* **95:**448-457.

2. Schmidt MK, Tollenaar RA, de K, Sr., Broeks A, Cornelisse CJ, Smit VT et al. (2007) **Breast cancer survival and tumor characteristics in premenopausal women carrying the CHEK2*1100delC germline mutation.** *J Clin Oncol* **25:**64-69.

3. Schrauder M, Frank S, Strissel PL, Lux MP, Bani MR et al. (2008) **Single nucleotide polymorphism D1853N of the ATM gene may alter the risk for breast cancer.** *J Cancer Res Clin Oncol* **134:**873-882.

4. Fletcher O, Johnson N, Palles C, Dos SS, I, McCormack V et al. (2006) **Inconsistent association between the STK15 F31I genetic polymorphism and breast cancer risk.** *J Natl Cancer Inst* **98:**1014-1018.

5. Colleran G, McInerney N, Rowan A, Barclay E, Jones AM et al. (2010) **The TGFBR1*6A/9A polymorphism is not associated with differential risk of breast cancer.** *Breast Cancer Res Treat* **119:**437-442.

6. Marme F, Werft W, Benner A, Burwinkel B, Sinn P et al. (2010) **FGFR4 Arg388 genotype is associated with pathological complete response to neoadjuvant chemotherapy for primary breast cancer.** *Ann Oncol* **21:**1636-1642.

7. Yang R, Dick M, Marme F, Schneeweiss A, Langheinz A et al. (2011) **Genetic variants within miR-126 and miR-335 are not associated with breast cancer risk.** *Breast Cancer Res Treat* **127:**549-554.

8. Villeneuve S, Fevotte J, Anger A, Truong T, Lamkarkach F et al. (2011) **Breast cancer risk by occupation and industry: Analysis of the CECILE study, a population-based case-control study in France.** *Am J Ind Med* **54(7):**499-509.

9. Weischer M, Bojesen SE, Tybjaerg-Hansen A, Axelsson CK, Nordestgaard BG (2007) **Increased risk of breast cancer associated with CHEK2*1100delC.** *J Clin Oncol* **25:**57-63.

10. Milne RL, Ribas G, Gonzalez-Neira A, Fagerholm R, Salas A et al. (2006) **ERCC4 associated with breast cancer risk: a two-stage case-control study using high-throughput genotyping.** *Cancer Res* **66:**9420-9427.

11. Widschwendter M, Apostolidou S, Raum E, Rothenbacher D, Fiegl H et al. (2008) **Epigenotyping in peripheral blood cell DNA and breast cancer risk: a proof of principle study.** *PLoS One* **3:**e2656.

12. Seal S, Thompson D, Renwick A, Elliott A, Kelly P et al. (2006) **Truncating mutations in the Fanconi anemia J gene BRIP1 are low-penetrance breast cancer susceptibility alleles.** *Nat Genet* **38:**1239-1241.

13. Frank B, Hemminki K, Wappenschmidt B, Meindl A, Klaes R et al. (2006) **Association of the CASP10 V410I variant with reduced familial breast cancer risk and interaction with the CASP8 D302H variant.** *Carcinogenesis* **27:**606-609.

14. Justenhoven C, Pierl CB, Haas S, Fischer HP, Baisch C et al. (2008) **The CYP1B1_1358_GG genotype is associated with estrogen receptor-negative breast cancer.** *Breast Cancer Res Treat* **111:**171-177.

15. Pesch B, Ko Y, Brauch H, Hamann U, Harth V et al. (2005) **Factors modifying the association between hormone-replacement therapy and breast cancer risk.** *Eur J Epidemiol* **20:**699-711.

16. Chang-Claude J, Eby N, Kiechle M, Bastert G, Becher H (2000) **Breastfeeding and breast cancer risk by age 50 among women in Germany.** *Cancer Causes Control* **11:**687-695.

17. Dork T, Bendix R, Bremer M, Rades D, Klopper K et al. (2001) **Spectrum of ATM gene mutations in a hospital-based series of unselected breast cancer patients.** *Cancer Res* **61:**7608-7615.

18. Fagerholm R, Hofstetter B, Tommiska J, Aaltonen K, Vrtel R, et al. (2008) **NAD(P)H:quinone oxidoreductase 1 NQO1*2 genotype (P187S) is a strong prognostic and predictive factor in breast cancer.** *Nat Genet* **40:**844-853.

19. Kilpivaara O, Bartkova J, Eerola H, Syrjakoski K, Vahteristo P et al. (2005) **Correlation of CHEK2 protein expression and c.1100delC mutation status with tumor characteristics among unselected breast cancer patients.** *Int J Cancer* **113:**575-580.

20. Syrjakoski K, Vahteristo P, Eerola H, Tamminen A, Kivinummi K et al. (2000) **Population-based study of BRCA1 and BRCA2 mutations in 1035 unselected Finnish breast cancer patients.** *J Natl Cancer Inst* **92:**1529-1531.

21. Bogdanova N, Cybulski C, Bermisheva M, Datsyuk I, Yamini P et al. (2009) **A nonsense mutation (E1978X) in the ATM gene is associated with breast cancer.** *Breast Cancer Res Treat* **118:**207-211.

22. Lindblom A, Rotstein S, Larsson C, Nordenskjold M, Iselius L (1992) **Hereditary breast cancer in Sweden: a predominance of maternally inherited cases.** *Breast Cancer Res Treat* **24:**159-165.

23. Margolin S, Werelius B, Fornander T, Lindblom A (2004) **BRCA1 mutations in a population-based study of breast cancer in Stockholm County.** *Genet Test* **8:**127-132.

24. Hartikainen JM, Tuhkanen H, Kataja V, Dunning AM, Antoniou A et al. (2005) **An autosome-wide scan for linkage disequilibrium-based association in sporadic breast cancer cases in eastern Finland: three candidate regions found.** *Cancer Epidemiol Biomarkers Prev* **14:**75-80.

25. Beesley J, Jordan SJ, Spurdle AB, Song H, Ramus SJ et al. (2007) **Association between single-nucleotide polymorphisms in hormone metabolism and DNA repair genes and epithelial ovarian cancer: results from two Australian studies and an additional validation set.** *Cancer Epidemiol Biomarkers Prev* **16:**2557-2565.

26. De ML, Van LE, De NK, Moerman P, Pochet N et al. (2008) **Does estrogen receptor negative/progesterone receptor positive breast carcinoma exist?** *J Clin Oncol* **26:**335-336.

27. Flesch-Janys D, Slanger T, Mutschelknauss E, Kropp S, Obi N et al. (2008) **Risk of different histological types of postmenopausal breast cancer by type and regimen of menopausal hormone therapy.** *Int J Cancer* **123:**933-941.

28. Catucci I, Verderio P, Pizzamiglio S, Manoukian S, Peissel B et al. (2009) **SNPs in ultraconserved elements and familial breast cancer risk.** *Carcinogenesis* **30:**544-545.

29. Olson JE, Ma CX, Pelleymounter LL, Schaid DJ, Pankratz VS, et al. (2007) **A comprehensive examination of CYP19 variation and breast density.** *Cancer Epidemiol Biomarkers Prev* **16:**623-625.

30. Giles GG, English DR (2002) **The Melbourne Collaborative Cohort Study.** *IARC Sci Publ* **156:**69-70.

31. Comen E, Balistreri L, Gonen M, Dutra-Clarke A, Fazio M et al. (2011) **Discriminatory accuracy and potential clinical utility of genomic profiling for breast cancer risk in BRCA-negative women.** *Breast Cancer Res Treat* **127:**479-487.

32. Nordgard SH, Johansen FE, Alnaes GI, Bucher E, Syvanen AC et al. (2008) **Genome-wide analysis identifies 16q deletion associated with survival, molecular subtypes, mRNA expression, and germline haplotypes in breast cancer patients.** *Genes Chromosomes Cancer* **47:**680-696.

33. John EM, Hopper JL, Beck JC, Knight JA, Neuhausen SL et al. (2004) **The Breast Cancer Family Registry: an infrastructure for cooperative multinational, interdisciplinary and translational studies of the genetic epidemiology of breast cancer.** *Breast Cancer Res* **6:**R375-R389.

34. Garcia-Closas M, Brinton LA, Lissowska J, Chatterjee N, Peplonska B et al. (2006) **Established breast cancer risk factors by clinically important tumour characteristics.** *Br J Cancer* **95:**123-129.

35. Easton DF, Pooley KA, Dunning AM, Pharoah PD, Thompson D et al. (2007) **Genome-wide association study identifies novel breast cancer susceptibility loci.** *Nature* **447:**1087-1093.

36. Wedren S, Lovmar L, Humphreys K, Magnusson C, Melhus H et al. (2004) **Oestrogen receptor alpha gene haplotype and postmenopausal breast cancer risk: a case control study.** *Breast Cancer Res* **6:**R437-R449.

37. MacPherson G, Healey CS, Teare MD, Balasubramanian SP, Reed MW et al. (2004) **Association of a common variant of the CASP8 gene with reduced risk of breast cancer.** *J Natl Cancer Inst* **96:**1866-1869.

38. Lesueur F, Pharoah PD, Laing S, Ahmed S, Jordan C et al. (2005) **Allelic association of the human homologue of the mouse modifier Ptprj with breast cancer.** *Hum Mol Genet* **14:**2349-2356.

39. Han S, Lee KM, Choi JY, Park SK, Lee JY et al. (2008) **CASP8 polymorphisms, estrogen and progesterone receptor status, and breast cancer risk.** *Breast Cancer Res Treat* **110:**387-393.

40. Lee KM, Choi JY, Park SK, Chung HW, Ahn B et al. (2005) **Genetic polymorphisms of ataxia telangiectasia mutated and breast cancer risk.** *Cancer Epidemiol Biomarkers Prev* **14:**821-825.

41. Jakubowska A, Jaworska K, Cybulski C, Janicka A, Szymanska-Pasternak J et al. (2009) **Do BRCA1 modifiers also affect the risk of breast cancer in non-carriers?** *Eur J Cancer* **45:**837-842.

42. Sangrajrang S, Schmezer P, Burkholder I, Waas P, Boffetta P et al. (2008) **Polymorphisms in three base excision repair genes and breast cancer risk in Thai women.** *Breast Cancer Res Treat* **111:**279-288.

43. Ding SL, Yu JC, Chen ST, Hsu GC, Kuo SJ et al. (2009) **Genetic variants of BLM interact with RAD51 to increase breast cancer susceptibility.** *Carcinogenesis* **30:**43-49.

44. Hsu HM, Wang HC, Chen ST, Hsu GC, Shen CY, Yu JC (2007) **Breast cancer risk is associated with the genes encoding the DNA double-strand break repair Mre11/Rad50/Nbs1 complex.** *Cancer Epidemiol Biomarkers Prev* **16:**2024-2032.

45. Ziogas A, Gildea M, Cohen P, Bringman D, Taylor TH et al. (2000) **Cancer risk estimates for family members of a population-based family registry for breast and ovarian cancer.** *Cancer Epidemiol Biomarkers Prev* **9:**103-111.

46. Garcia-Closas M, Egan KM, Newcomb PA, Brinton LA, Titus-Ernstoff L et al. (2006) **Polymorphisms in DNA double-strand break repair genes and risk of breast cancer: two population-based studies in USA and Poland, and meta-analyses.** *Hum Genet* **119:**376-388.
