# Supplementary material for: Comparison of 6q25 Breast Cancer Hits from Asian and European Genome Wide Association Studies in the Breast Cancer Association Consortium (BCAC)
Source: PLoS One. 2012 Aug 7;7(8):e42380. doi: 10.1371/journal.pone.0042380 (PMC3413660; doi:10.1371/journal.pone.0042380)
Supplement: Table S2 — Characteristics of the study populations genotyped for rs2046210 (a) and rs12662670 (b). (DOC) [file pone.0042380.s002.doc]

Table S2a: Characteristics of the study populations genotyped for rs2046210.

| **Study** | **Genotyping phase** | **Genotyping call rate** | **Dupli-cate con-cor-dance** | **Coriell call rate** | **Coriell concor-dance** | **p-HWE Controls** | **Invasive cases** | **In-situ cases** | **ER+ cases** | **ER- cases** | **European ethnicity** | | **Asian ethnicity** | |
| --- | --- | --- | --- | --- | --- | --- | --- | --- | --- | --- | --- | --- | --- | --- |
|
|  |  |  |  |  |  |  |  |  |  |  | **No. Cases** | **No. Con-trols** | **No. Cases** | **No. Con-trols** |
| ABCFS | 9 | 99.00 | 100.00 | 100.00 | 100.00 | 0.97 | 1423 | 0 | 801 | 417 | 1307 | 699 | 63 | 17 |
| ABCS | 9 & 10 | 99.85 & 100 | 100.00 & 98.62 | 100.00 | 100.00 | 0.00004 & 0.01 | 2075 | 92 | 627 | 329 | 2475 | 974 | 0 | 0 |
| BBCC | 9 | 96.87 | 99.12 | 90.43 | 89.41 | 0.57 | 921 | 51 | 611 | 257 | 972 | 874 | 0 | 0 |
| BBCS | 9 | 99.90 | 100.00 | 98.94 | 100.00 | 0.45 | 1150 | 0 | 0 | 0 | 1150 | 826 | 0 | 0 |
| BIGGS | 9 | 98.60 | 100.00 | 100.00 | 100.00 | 0.77 | 832 | 47 | 488 | 166 | 878 | 825 | 1 | 0 |
| BSUCH | 9 | 99.10 | 100.00 | 100.00 | 100.00 | 0.28 | 1074 | 18 | 584 | 234 | 1092 | 930 | 0 | 0 |
| CECILE | 10 | 100.00 | 100.00 | 100.00 | 100.00 | 0.64 | 945 | 122 | 830 | 158 | 1060 | 1028 | 1 | 0 |
| CGPS | 9 | 98.90 | 100.00 | 94.68 | 100.00 | 0.32 | 2623 | 99 | 1924 | 407 | 2722 | 6542 | 0 | 3 |
| CNIO-BCS | 9 | 98.60 | 100.00 | 100.00 | 100.00 | 0.91 | 915 | 33 | 262 | 95 | 948 | 790 | 0 | 0 |
| ESTHER | 9 | 99.70 | 96.36 | 100.00 | 100.00 | 0.02 | 434 | 5 | 329 | 97 | 497 | 507 | 0 | 0 |
| FBCS | 9 | 98.70 | 100.00 | 100.00 | 100.00 | 0.07 | 1728 | 0 | 0 | 0 | 1728 | 1043 | 0 | 0 |
| GENICA | 9 | 99.70 | 100.00 | 98.94 | 100.00 | 0.13 | 957 | 0 | 712 | 198 | 957 | 975 | 0 | 0 |
| GESBC | 9 | 99.40 | 100.00 | 98.94 | 100.00 | 0.86 | 503 | 36 | 252 | 157 | 547 | 554 | 0 | 0 |
| HABCS | 9 | 100.00 | 100.00 | 95.74 | 98.89 | 0.12 | 1008 | 13 | 615 | 74 | 1015 | 984 | 4 | 10 |
| HEBCS | 9 | 99.80 | 100.00 | 100.00 | 100.00 | 0.43 | 2217 | 152 | 1777 | 422 | 2369 | 1250 | 0 | 0 |
| HMBCS | 9 | 100.00 | 100.00 | 95.74 | 98.89 | 0.32 | 1649 | 0 | 0 | 0 | 1649 | 913 | 0 | 0 |
| HUBCS | 9 | 99.90 | 100.00 | 95.74 | 98.89 | 0.06 | 938 | 0 | 110 | 86 | 878 | 1360 | 60 | 54 |
| KARBAC | 9 | 99.50 | 100.00 | 100.00 | 100.00 | 0.68 | 805 | 0 | 355 | 73 | 792 | 839 | 5 | 0 |
| KBCP | 9 | 99.30 | 100.00 | 100.00 | 98.94 | 0.76 | 466 | 22 | 335 | 103 | 488 | 397 | 0 | 0 |
| kConFab/ AOCS | 9 | 99.90 | 100.00 | 100.00 | 100.00 | 0.91 | 383 | 103 | 169 | 73 | 523 | 903 | 2 | 9 |
| LMBC | 9 | 100.00 | 99.50 | 100.00 | 98.94 | 0.65 | 2714 | 209 | 2263 | 414 | 2925 | 1584 | 0 | 0 |
| MBCSG | 9 | 99.50 | 100.00 | 100.00 | 100.00 | 0.01 | 304 | 28 | 221 | 68 | 743 | 1342 | 0 | 0 |
| MCBCS | 9 | 97.90 | 100.00 | 100.00 | 98.94 | 0.65 | 1367 | 266 | 1320 | 259 | 1633 | 1415 | 0 | 0 |
| MCCS | 9 | 99.60 | 100.00 | 100.00 | 100.00 | 0.48 | 668 | 0 | 410 | 132 | 668 | 758 | 0 | 0 |
| MSKCC | 9 | 99.61 | 100.00 | 100.00 | 100.00 | 0.63 | 535 | 0 | 0 | 0 | 491 | 455 | 7 | 4 |
| NBCS | 9 | 98.83 | 95.75 | 60.64 | 91.23 | 0.96 | 655 | 0 | 262 | 164 | 1632 | 1869 | 2 | 0 |
| NC-BCFR | 9 | 99.80 | 98.90 | 100.00 | 100.00 | 0.10 | 1386 | 226 | 903 | 316 | 386 | 154 | 459 | 61 |
| OBCS | 9 | 100.00 | 100.00 | 100.00 | 98.94 | 0.75 | 530 | 7 | 430 | 107 | 537 | 497 | 0 | 0 |
| OFBCR | 9 | 100.00 | 100.00 | 98.94 | 100.00 | 0.44 | 1370 | 17 | 694 | 283 | 1153 | 328 | 123 | 15 |
| PBCS | 9 | 99.44 | 100.00 | 100.00 | 100.00 | 0.67 | 1870 | 127 | 1145 | 595 | 2083 | 2288 | 0 | 0 |
| RBCS | 9 | 99.60 | 100.00 | 100.00 | 100.00 | 0.09 | 698 | 44 | 401 | 148 | 744 | 785 | 0 | 0 |
| SASBAC | 9 | 99.90 | 100.00 | 100.00 | 100.00 | 0.47 | 1199 | 0 | 691 | 149 | 1199 | 1358 | 0 | 0 |
| SBCS | 9 | 99.40 | 100.00 | 100.00 | 100.00 | 0.55 | 869 | 68 | 439 | 118 | 966 | 948 | 0 | 0 |
| SEARCH | 9 | 99.20 | 99.30 | 100.00 | 100.00 | 0.89 | 6507 | 0 | 2472 | 578 | 6441 | 6672 | 36 | 10 |
| SEBCS | 9 | 96.40 | 100.00 | 100.00 | 100.00 | 0.06 | 1661 | 0 | 925 | 545 | 0 | 0 | 1661 | 1138 |
| SZBCS | 9 | 99.30 | 100.00 | 98.94 | 100.00 | 0.78 | 663 | 32 | 525 | 210 | 848 | 800 | 0 | 0 |
| TBCS | 9 | 97.00 | 100.00 | 95.74 | 94.44 | 0.37 | 431 | 0 | 111 | 110 | 0 | 0 | 431 | 292 |
| TWBCS | 9 | 99.60 | 100.00 | 98.94 | 98.92 | 0.10 | 862 | 29 | 271 | 152 | 0 | 0 | 891 | 904 |
| UCIBCS | 9 | 99.00 | 100.00 | 100.00 | 100.00 | 0.49 | 895 | 148 | 594 | 162 | 893 | 496 | 53 | 15 |
| UKBGS | 9 | 98.36 | 94.23 | 98.94 | 100.00 | 0.96 | 2231 | 76 | 0 | 0 | 2297 | 2356 | 11 | 7 |
| US3SS | 9 | 95.18 | 97.78 | 100.00 | 100.00 | 0.27 | 1433 | 202 | 0 | 0 | 1580 | 1230 | 0 | 0 |
| **Total** | **-** | **99.11** | **99.52** | **98.08** | **99.18** | **-** | **51894** | **2272** | **24858** | **7856** | **51266** | **48548** | **3810** | **2539** |

Table S2b: Characteristics of the study populations genotyped for rs12662670.

| **Study** | **Genotyping phase** | **Genotyping call rate** | **Dupli-cate con-cor-dance** | **Coriell call rate** | **Coriell concor-dance** | **p-HWE Controls** | **Invasive cases** | **In-situ cases** | **ER+ cases** | **ER- cases** | **European ethnicity** | | **Asian ethnicity** | |
| --- | --- | --- | --- | --- | --- | --- | --- | --- | --- | --- | --- | --- | --- | --- |
|
|  |  |  |  |  |  |  |  |  |  |  | **No. Cases** | **No. Con-trols** | **No. Cases** | **No. Con-trols** |
| ABCS | 11 | 99.71 | 100.00 | 0.00 | 0.00 | 0.56 | 687 | 62 | 0 | 0 | 1090 | 972 | 0 | 0 |
| ACP | 11 | 99.60 | 98.46 | 0.00 | 0.00 | 0.95 | 324 | 0 | 0 | 0 | 0 | 0 | 324 | 561 |
| BBCC | 11 | 99.74 | 100.00 | 0.00 | 0.00 | 0.17 | 1222 | 55 | 830 | 328 | 1276 | 603 | 1 | 0 |
| BBCS | 11 | 99.95 | 100.00 | 0.00 | 0.00 | 0.64 | 1149 | 0 | 0 | 0 | 1149 | 827 | 0 | 0 |
| BIGGS | 11 | 99.83 | 100.00 | 0.00 | 0.00 | 0.05 | 868 | 50 | 512 | 171 | 917 | 832 | 1 | 0 |
| BSUCH | 11 | 99.83 | 100.00 | 100.00 | 100.00 | 0.32 | 1075 | 19 | 583 | 237 | 1094 | 1306 | 0 | 0 |
| CNIO-BCS | 11 | 99.72 | 100.00 | 0.00 | 0.00 | 0.21 | 845 | 33 | 253 | 93 | 878 | 779 | 0 | 0 |
| CTS | 11 | 100.00 | 100.00 | 0.00 | 0.00 | 0.85 | 1372 | 0 | 0 | 0 | 1253 | 1226 | 42 | 31 |
| ESTHER | 11 | 100.00 | 100.00 | 0.00 | 0.00 | 0.17 | 437 | 5 | 329 | 100 | 500 | 504 | 0 | 0 |
| GC-HBOC | 11 | 99.80 | 100.00 | 0.00 | 0.00 | 0.76 | 847 | 0 | 0 | 0 | 847 | 1124 | 0 | 0 |
| GENICA | 11 | 99.54 | 100.00 | 100.00 | 100.00 | 0.02 | 952 | 0 | 708 | 199 | 952 | 978 | 0 | 0 |
| GESBC | 11 | 100.00 | 100.00 | 0.00 | 0.00 | 0.72 | 433 | 32 | 218 | 131 | 472 | 553 | 0 | 0 |
| HABCS | 10 | 99.69 | 99.45 | 100.00 | 100.00 | 0.11 | 982 | 13 | 599 | 70 | 989 | 948 | 4 | 10 |
| HEBCS | 11 | 99.86 | 100.00 | 0.00 | 0.00 | 0.81 | 2206 | 152 | 1771 | 417 | 2358 | 1252 | 0 | 0 |
| HMBCS | 10 | 99.49 | 99.09 | 100.00 | 100.00 | 0.01 | 1641 | 0 | 0 | 0 | 1641 | 902 | 0 | 0 |
| HUBCS | 10 | 99.37 | 100.00 | 100.00 | 100.00 | 0.01 | 936 | 0 | 110 | 85 | 877 | 1341 | 59 | 54 |
| KARBAC | 10 | 99.88 | 100.00 | 100.00 | 100.00 | 0.53 | 795 | 0 | 354 | 72 | 782 | 801 | 5 | 0 |
| KBCP | 11 | 100.00 | 100.00 | 100.00 | 100.00 | 0.04 | 462 | 22 | 332 | 102 | 484 | 357 | 0 | 0 |
| kConFab/ AOCS | 11 | 100.00 | 100.00 | 0.00 | 0.00 | 0.71 | 451 | 112 | 156 | 67 | 622 | 938 | 3 | 10 |
| LMBC | 11 | 99.89 | 100.00 | 100.00 | 100.00 | 0.37 | 2718 | 210 | 2274 | 412 | 2930 | 1596 | 0 | 0 |
| MARIE | 11 | 99.89 | 99.84 | 0.00 | 0.00 | 0.57 | 2382 | 164 | 1910 | 531 | 2543 | 4896 | 1 | 6 |
| MBCSG | 11 | 100.00 | 100.00 | 0.00 | 0.00 | 0.15 | 307 | 29 | 223 | 69 | 748 | 1344 | 0 | 0 |
| MCBCS | 11 | 100.00 | 100.00 | 95.74 | 100.00 | 0.78 | 1478 | 287 | 1429 | 274 | 1765 | 1282 | 0 | 0 |
| NBCS | 11 | 99.77 | 99.61 | 81.00 | 100.00 | 0.45 | 662 | 0 | 266 | 168 | 1620 | 1813 | 2 | 0 |
| OBCS | 11 | 99.90 | 100.00 | 0.00 | 0.00 | 0.54 | 530 | 7 | 430 | 107 | 537 | 495 | 0 | 0 |
| OFBCR | 11 | 99.77 | 100.00 | 100.00 | 100.00 | 0.07 | 1307 | 17 | 662 | 267 | 1098 | 320 | 120 | 15 |
| RBCS | 10 | 99.94 | 100.00 | 100.00 | 100.00 | 0.43 | 702 | 44 | 406 | 148 | 748 | 786 | 0 | 0 |
| SASBAC | 11 | 99.71 | 100.00 | 0.00 | 0.00 | 0.77 | 1224 | 0 | 700 | 155 | 1224 | 1464 | 0 | 0 |
| SBCS | 11 | 99.79 | 100.00 | 0.00 | 0.00 | 0.52 | 875 | 69 | 442 | 118 | 973 | 949 | 0 | 0 |
| SEARCH | 10 | 99.78 | 100.00 | 100.00 | 100.00 | 0.90 | 6532 | 0 | 2483 | 582 | 6466 | 6719 | 36 | 10 |
| SEBCS | 10 | 99.43 | 99.44 | 100.00 | 100.00 | 0.38 | 2070 | 0 | 1129 | 657 | 0 | 0 | 2070 | 996 |
| SZBCS | 11 | 99.94 | 100.00 | 100.00 | 100.00 | 0.38 | 616 | 31 | 496 | 198 | 782 | 892 | 0 | 0 |
| TWBCS | 11 | 98.74 | 100.00 | 93.62 | 100.00 | 0.67 | 850 | 29 | 265 | 151 | 0 | 0 | 879 | 894 |
| UCIBCS | 11 | 99.93 | 100.00 | 0.00 | 0.00 | 0.25 | 743 | 113 | 491 | 137 | 728 | 414 | 46 | 12 |
| **Total** | **-** | **99.78** | **99.88** | **46.19** | **47.06** | **-** | **40680** | **1555** | **20361** | **6046** | **40343** | **39213** | **3593** | **2599** |
