# Supplementary material for: Comparison of 6q25 Breast Cancer Hits from Asian and European Genome Wide Association Studies in the Breast Cancer Association Consortium (BCAC)
Source: PLoS One. 2012 Aug 7;7(8):e42380. doi: 10.1371/journal.pone.0042380 (PMC3413660; doi:10.1371/journal.pone.0042380)
Supplement: Table S3 — Genotype frequencies of SNP rs2046210 (a) and rs12662670 (b) in the different studies. (DOC) [file pone.0042380.s003.doc]

**Table S3**a: Genotype frequencies of SNP rs2046210 in the different studies.

| **Study** | **MAF^a^** | | **Genotype GG** | | **Genotype GA** | | **Genotype AA** | |
| --- | --- | --- | --- | --- | --- | --- | --- | --- |
|  | **Cases** | **Controls** | **No. (%) Cases** | **No. (%) Controls** | **No. (%) Cases** | **No. (%) Controls** | **No. (%) Cases** | **No. (%) Controls** |
| ABCFS^b^ | 0.39 | 0.36 | 548 (0.39) | 317 (0.42) | 667 (0.47) | 337 (0.45) | 208 (0.15) | 101 (0.13) |
| ABCS^b^ | 0.37 | 0.34 | 990 (0.4) | 446 (0.46) | 1170 (0.47) | 402 (0.41) | 315 (0.13) | 126 (0.13) |
| BBCC^b^ | 0.32 | 0.34 | 430 (0.44) | 388 (0.44) | 467 (0.48) | 380 (0.43) | 75 (0.08) | 106 (0.12) |
| BBCS^b^ | 0.38 | 0.36 | 437 (0.38) | 340 (0.41) | 547 (0.48) | 388 (0.47) | 166 (0.14) | 98 (0.12) |
| BIGGS^b^ | 0.38 | 0.36 | 334 (0.38) | 339 (0.41) | 423 (0.48) | 384 (0.47) | 122 (0.14) | 102 (0.12) |
| BSUCH^b^ | 0.36 | 0.36 | 438 (0.4) | 378 (0.41) | 527 (0.48) | 443 (0.48) | 127 (0.12) | 109 (0.12) |
| CECILE^b^ | 0.40 | 0.37 | 371 (0.35) | 421 (0.41) | 538 (0.5) | 474 (0.46) | 158 (0.15) | 142 (0.14) |
| CGPS^b^ | 0.35 | 0.34 | 1152 (0.42) | 2825 (0.43) | 1235 (0.45) | 2980 (0.46) | 335 (0.12) | 743 (0.11) |
| CNIO-BCS^b^ | 0.40 | 0.39 | 332 (0.35) | 297 (0.38) | 474 (0.5) | 372 (0.47) | 142 (0.15) | 121 (0.15) |
| ESTHER^b^ | 0.36 | 0.34 | 208 (0.42) | 234 (0.46) | 226 (0.45) | 204 (0.4) | 63 (0.13) | 69 (0.14) |
| FBCS^b^ | 0.38 | 0.36 | 694 (0.4) | 447 (0.43) | 778 (0.45) | 455 (0.44) | 256 (0.15) | 141 (0.14) |
| GENICA^b^ | 0.35 | 0.34 | 412 (0.43) | 442 (0.45) | 428 (0.45) | 415 (0.43) | 117 (0.12) | 118 (0.12) |
| GESBC^b^ | 0.38 | 0.32 | 221 (0.4) | 264 (0.48) | 241 (0.44) | 237 (0.43) | 85 (0.16) | 53 (0.1) |
| HABCS^b^ | 0.33 | 0.32 | 453 (0.44) | 455 (0.46) | 465 (0.46) | 451 (0.45) | 103 (0.1) | 91 (0.09) |
| HEBCS^b^ | 0.25 | 0.23 | 1367 (0.58) | 748 (0.6) | 843 (0.36) | 444 (0.36) | 159 (0.07) | 58 (0.05) |
| HMBCS^b^ | 0.29 | 0.29 | 851 (0.52) | 471 (0.52) | 673 (0.41) | 373 (0.41) | 125 (0.08) | 69 (0.08) |
| HUBCS^b^ | 0.28 | 0.27 | 486 (0.52) | 778 (0.55) | 375 (0.4) | 524 (0.37) | 77 (0.08) | 112 (0.08) |
| KARBAC^b^ | 0.34 | 0.33 | 351 (0.44) | 381 (0.45) | 356 (0.44) | 367 (0.44) | 98 (0.12) | 91 (0.11) |
| KBCP^b^ | 0.23 | 0.21 | 296 (0.61) | 253 (0.64) | 170 (0.35) | 126 (0.32) | 22 (0.05) | 18 (0.05) |
| kConFab/ AOCS^b^ | 0.38 | 0.35 | 210 (0.38) | 398 (0.42) | 269 (0.48) | 428 (0.45) | 79 (0.14) | 116 (0.12) |
| LMBC^b^ | 0.37 | 0.34 | 1158 (0.4) | 689 (0.43) | 1378 (0.47) | 718 (0.45) | 389 (0.13) | 177 (0.11) |
| MBCSG^b^ | 0.40 | 0.37 | 262 (0.35) | 508 (0.38) | 360 (0.48) | 671 (0.5) | 121 (0.16) | 163 (0.12) |
| MCBCS^b^ | 0.35 | 0.36 | 668 (0.41) | 607 (0.43) | 780 (0.48) | 631 (0.45) | 185 (0.11) | 177 (0.13) |
| MCCS^b^ | 0.40 | 0.37 | 236 (0.35) | 315 (0.42) | 322 (0.48) | 340 (0.45) | 110 (0.16) | 103 (0.14) |
| MSKCC^b^ | 0.43 | 0.38 | 177 (0.33) | 185 (0.39) | 258 (0.48) | 230 (0.48) | 100 (0.19) | 65 (0.14) |
| NC-BCFR^b^ | 0.42 | 0.39 | 577 (0.36) | 133 (0.4) | 729 (0.45) | 144 (0.43) | 306 (0.19) | 57 (0.17) |
| OBCS^b^ | 0.22 | 0.20 | 326 (0.61) | 318 (0.64) | 188 (0.35) | 161 (0.32) | 23 (0.04) | 18 (0.04) |
| OFBCR^b^ | 0.38 | 0.33 | 555 (0.4) | 162 (0.44) | 622 (0.45) | 167 (0.46) | 210 (0.15) | 36 (0.1) |
| PBCS^b^ | 0.32 | 0.29 | 970 (0.47) | 1136 (0.5) | 897 (0.43) | 959 (0.42) | 216 (0.1) | 193 (0.08) |
| RBCS^b^ | 0.36 | 0.31 | 299 (0.4) | 360 (0.46) | 363 (0.49) | 359 (0.46) | 82 (0.11) | 66 (0.08) |
| SASBAC^b^ | 0.36 | 0.33 | 507 (0.42) | 618 (0.46) | 537 (0.45) | 605 (0.45) | 155 (0.13) | 135 (0.1) |
| SBCS^b^ | 0.37 | 0.37 | 397 (0.41) | 378 (0.4) | 435 (0.45) | 446 (0.47) | 136 (0.14) | 124 (0.13) |
| SEARCH^b^ | 0.38 | 0.36 | 2517 (0.39) | 2761 (0.41) | 3062 (0.47) | 3078 (0.46) | 928 (0.14) | 851 (0.13) |
| SEBCS^c^ | 0.38 | 0.31 | 627 (0.38) | 560 (0.49) | 795 (0.48) | 461 (0.41) | 239 (0.14) | 117 (0.1) |
| SZBCS^b^ | 0.34 | 0.30 | 380 (0.45) | 391 (0.49) | 366 (0.43) | 336 (0.42) | 102 (0.12) | 73 (0.09) |
| TBCS^c^ | 0.47 | 0.41 | 120 (0.28) | 100 (0.34) | 220 (0.51) | 148 (0.51) | 91 (0.21) | 44 (0.15) |
| TWBCS^c^ | 0.43 | 0.37 | 294 (0.33) | 360 (0.4) | 440 (0.49) | 439 (0.49) | 157 (0.18) | 105 (0.12) |
| UCIBCS^b^ | 0.37 | 0.37 | 427 (0.41) | 231 (0.41) | 459 (0.44) | 249 (0.45) | 157 (0.15) | 78 (0.14) |
| UKBGS^b^ | 0.35 | 0.34 | 1003 (0.43) | 1035 (0.44) | 1006 (0.44) | 1059 (0.45) | 303 (0.13) | 272 (0.11) |
| US3SS^b^ | 0.37 | 0.36 | 636 (0.39) | 505 (0.4) | 786 (0.48) | 604 (0.48) | 213 (0.13) | 158 (0.12) |
| **Total** | **0.36** | **0.33** | **23398 (0.42)** | **22843 (0.44)** | **25645 (0.46)** | **22799 (0.44)** | **7238 (0.13)** | **5786 (0.11)** |

^a^MAF: minor allele frequency of A allele

^b^Europeans

^c^Asians

**Table S3**b: Genotype frequencies of SNP rs12662670 in the different studies.

| **Study** | **MAF^a^** | | **Genotype TT** | | **Genotype GT** | | **Genotype GG** | |
| --- | --- | --- | --- | --- | --- | --- | --- | --- |
|  | **Cases** | **Controls** | **No. (%) Cases** | **No. (%) Controls** | **No. (%) Cases** | **No. (%) Controls** | **No. (%) Cases** | **No. (%) Controls** |
| ABCS^b^ | 0.10 | 0.09 | 892 (0.82) | 808 (0.83) | 189 (0.17) | 159 (0.16) | 9 (0.01) | 5 (0.01) |
| ACP^c^ | 0.35 | 0.33 | 137 (0.42) | 253 (0.45) | 144 (0.44) | 247 (0.44) | 43 (0.13) | 61 (0.11) |
| BBCC^b^ | 0.08 | 0.08 | 1087 (0.85) | 510 (0.85) | 181 (0.14) | 86 (0.14) | 9 (0.01) | 7 (0.01) |
| BBCS^b^ | 0.10 | 0.08 | 941 (0.82) | 705 (0.85) | 202 (0.18) | 116 (0.14) | 6 (0.01) | 6 (0.01) |
| BIGGS^b^ | 0.09 | 0.08 | 757 (0.82) | 714 (0.86) | 151 (0.16) | 109 (0.13) | 10 (0.01) | 9 (0.01) |
| BSUCH^b^ | 0.09 | 0.08 | 911 (0.83) | 1101 (0.84) | 174 (0.16) | 199 (0.15) | 9 (0.01) | 6 (0) |
| CNIO-BCS^b^ | 0.09 | 0.09 | 725 (0.83) | 643 (0.83) | 144 (0.16) | 126 (0.16) | 9 (0.01) | 10 (0.01) |
| CTS^b^ | 0.10 | 0.09 | 1124 (0.82) | 1100 (0.84) | 232 (0.17) | 206 (0.16) | 16 (0.01) | 9 (0.01) |
| ESTHER^b^ | 0.08 | 0.08 | 423 (0.85) | 425 (0.84) | 71 (0.14) | 73 (0.14) | 6 (0.01) | 6 (0.01) |
| GC-HBOC^b^ | 0.10 | 0.09 | 696 (0.82) | 944 (0.84) | 143 (0.17) | 173 (0.15) | 8 (0.01) | 7 (0.01) |
| GENICA^b^ | 0.10 | 0.08 | 786 (0.83) | 819 (0.84) | 158 (0.17) | 158 (0.16) | 8 (0.01) | 1 (0) |
| GESBC^b^ | 0.07 | 0.07 | 407 (0.86) | 480 (0.87) | 63 (0.13) | 71 (0.13) | 2 (0) | 2 (0) |
| HABCS^b^ | 0.08 | 0.07 | 851 (0.86) | 834 (0.87) | 139 (0.14) | 119 (0.12) | 5 (0.01) | 8 (0.01) |
| HEBCS^b^ | 0.04 | 0.04 | 2162 (0.92) | 1148 (0.92) | 191 (0.08) | 102 (0.08) | 5 (0) | 2 (0) |
| HMBCS^b^ | 0.05 | 0.06 | 1467 (0.89) | 809 (0.9) | 169 (0.1) | 86 (0.1) | 5 (0) | 7 (0.01) |
| HUBCS^b^ | 0.08 | 0.07 | 797 (0.85) | 1205 (0.86) | 133 (0.14) | 174 (0.12) | 6 (0.01) | 16 (0.01) |
| KARBAC^b^ | 0.08 | 0.07 | 678 (0.85) | 688 (0.86) | 113 (0.14) | 110 (0.14) | 4 (0.01) | 3 (0) |
| KBCP^b^ | 0.04 | 0.05 | 443 (0.92) | 331 (0.93) | 40 (0.08) | 24 (0.07) | 1 (0) | 2 (0.01) |
| kConFab/ AOCS^b^ | 0.10 | 0.08 | 536 (0.82) | 842 (0.86) | 113 (0.17) | 131 (0.13) | 7 (0.01) | 6 (0.01) |
| LMBC^b^ | 0.10 | 0.09 | 2369 (0.81) | 1327 (0.83) | 534 (0.18) | 260 (0.16) | 27 (0.01) | 9 (0.01) |
| MARIE^b^ | 0.09 | 0.08 | 2144 (0.84) | 4191 (0.85) | 386 (0.15) | 682 (0.14) | 16 (0.01) | 31 (0.01) |
| MBCSG^b^ | 0.11 | 0.10 | 591 (0.79) | 1082 (0.81) | 147 (0.2) | 242 (0.18) | 10 (0.01) | 20 (0.01) |
| MCBCS^b^ | 0.09 | 0.08 | 1467 (0.83) | 1094 (0.85) | 293 (0.17) | 181 (0.14) | 5 (0) | 7 (0.01) |
| OBCS^b^ | 0.05 | 0.03 | 497 (0.93) | 461 (0.93) | 36 (0.07) | 32 (0.06) | 4 (0.01) | 2 (0) |
| OFBCR^b^ | 0.10 | 0.08 | 1088 (0.82) | 302 (0.85) | 222 (0.17) | 49 (0.14) | 14 (0.01) | 5 (0.01) |
| RBCS^b^ | 0.09 | 0.07 | 623 (0.83) | 670 (0.85) | 115 (0.15) | 113 (0.14) | 10 (0.01) | 3 (0) |
| SASBAC^b^ | 0.07 | 0.06 | 1044 (0.85) | 1279 (0.87) | 174 (0.14) | 178 (0.12) | 6 (0) | 7 (0) |
| SBCS^b^ | 0.09 | 0.08 | 811 (0.83) | 821 (0.87) | 152 (0.16) | 122 (0.13) | 12 (0.01) | 6 (0.01) |
| SEARCH^b^ | 0.09 | 0.09 | 5414 (0.83) | 5706 (0.85) | 1065 (0.16) | 988 (0.15) | 53 (0.01) | 43 (0.01) |
| SEBCS^c^ | 0.33 | 0.27 | 941 (0.45) | 530 (0.53) | 897 (0.43) | 400 (0.4) | 232 (0.11) | 66 (0.07) |
| SZBCS^b^ | 0.07 | 0.05 | 674 (0.86) | 803 (0.9) | 107 (0.14) | 88 (0.1) | 1 (0) | 1 (0) |
| TWBCS^c^ | 0.36 | 0.29 | 367 (0.42) | 441 (0.49) | 400 (0.46) | 378 (0.42) | 112 (0.13) | 75 (0.08) |
| UCIBCS^b^ | 0.10 | 0.08 | 695 (0.81) | 396 (0.85) | 149 (0.17) | 64 (0.14) | 12 (0.01) | 5 (0.01) |
| **Total** | 0.11 | 0.09 | **35932 (0.81)** | **35027 (0.83)** | **7651 (0.17)** | **6487 (0.15)** | **693 (0.02)** | **460 (0.01)** |

^a^MAF: minor allele frequency of G allele

^b^Europeans

^c^Asians
