# Supplementary material for: Comparison of 6q25 Breast Cancer Hits from Asian and European Genome Wide Association Studies in the Breast Cancer Association Consortium (BCAC)
Source: PLoS One. 2012 Aug 7;7(8):e42380. doi: 10.1371/journal.pone.0042380 (PMC3413660; doi:10.1371/journal.pone.0042380)
Supplement: Table S4 — Association of rs2046210 and rs12662670 with in-situ/invasive breast cancer. (DOC) [file pone.0042380.s004.doc]

**Table S4: Association of rs2046210 and rs12662670** with in-situ / invasive breast cancer.

| **Ethnicity** | **Type of tumour** | **Number of cases/controls** | **OR (95% confidence interval)^a^** | **P-value^b^** |
| --- | --- | --- | --- | --- |
| **rs2046210** |  |  |  |  |
| *Europeans* | *in-situ* | 1318 / 9445 | 1.15 (1.05-1.25) | 1.85x10^-3^ |
|  | *invasive* | 46,405 / 46,679 | 1.09 (1.07-1.11) | 1.68x10^-16^ |
| *Asians* | *invasive* | 2954 / 2334 | 1.36 (1.26-1.48) | 7.54x10^-14^ |
| **rs12662670** |  |  |  |  |
| *Europeans* | *in-situ* | 917 / 9964 | 1.13 (0.94-1.35) | 1.87x10^-1^ |
|  | *invasive* | 36,152 / 37,400 | 1.12 (1.08-1.16) | 2.04x10^-8^ |
| *Asians* | *invasive* | 3244 / 2451 | 1.29 (1.19-1.40) | 1.74x10^-9^ |

Results are presented separately for Europeans and Asians. Pooled analyses adjusted for study were performed. Estimates and p-values were not calculated for in-situ cases and controls in Asians since data were too sparse (< 100 cases and/or controls per category).

^a^Odds ratio per minor allele (A allele for rs2046210, G allele for rs12662670).

^b^P-value derived from a log-additive model.
