# Supplementary material for: Comparison of 6q25 Breast Cancer Hits from Asian and European Genome Wide Association Studies in the Breast Cancer Association Consortium (BCAC)
Source: PLoS One. 2012 Aug 7;7(8):e42380. doi: 10.1371/journal.pone.0042380 (PMC3413660; doi:10.1371/journal.pone.0042380)
Supplement: Table S5 — Association of rs2046210 and rs12662670 with risk of ER−*/ER+**breast cancer. (DOC) [file pone.0042380.s005.doc]

**Table S5: Association of rs2046210 and rs12662670 with risk of ER-^*^ / ER+^**^** breast cancer.

| **Ethnicity** | **Estrogen receptor status** | **Age** | **Number of cases/controls** | **OR (95% confidence interval)^a^** | **P-value^b^** | **P-trend^c^** |
| --- | --- | --- | --- | --- | --- | --- |
| **rs2046210** |  |  |  |  |  |  |
| *Overall* | *ER-^*^* | *<40* | 255 / 387 | 1.26 (1.00-1.60) | 5.28x10^-2^ |  |
|  |  | *40-49* | 788 / 2378 | 1.24 (1.10-1.40) | 6.53x10^-4^ |  |
|  |  | *50-59* | 906 / 4238 | 1.28 (1.14-1.42) | 1.80x10^-5^ |  |
|  |  | *≥60* | 644 / 6388 | 1.12 (0.99-1.28) | 8.42x10^-2^ | 1.84x10^-2^ |
|  | *ER+^**^* | *<40* | 819 / 2213 | 1.19 (1.04-1.34) | 8.65x10^-3^ |  |
|  |  | *40-49* | 4181 / 5868 | 1.07 (1.01-1.14) | 3.51x10^-2^ |  |
|  |  | *50-59* | 6579 / 9771 | 1.12 (1.06-1.18) | 1.08x10^-5^ |  |
|  |  | *≥60* | 6635 / 11289 | 1.05 (1.00-1.10) | 4.36x10^-2^ | 5.59x10^-5^ |
| *Europeans* | *ER-^*^* | *<40* | 232 / 344 | 1.26 (0.98-1.61) | 7.34x10^-2^ |  |
|  |  | *40-49* | 784 / 2374 | 1.25 (1.10-1.41) | 4.81x10^-4^ |  |
|  |  | *50-59* | 775 / 4054 | 1.27 (1.13-1.43) | 6.93x10^-5^ |  |
|  |  | *≥60* | 644 / 6385 | 1.12 (0.98-1.28) | 8.42x10^-2^ | 1.34x10^-2^ |
|  | *ER+^**^* | *<40* | 792 / 2170 | 1.19 (1.05-1.36) | 6.82x10^-3^ |  |
|  |  | *40-49* | 4126 / 5833 | 1.07 (1.01-1.14) | 3.40x10^-2^ |  |
|  |  | *50-59* | 6250 / 9669 | 1.12 (1.06-1.18) | 1.45x10^-5^ |  |
|  |  | *≥60* | 6594 / 11265 | 1.05 (1.00-1.10) | 4.53x10^-2^ | 7.24x10^-5^ |
| **rs12662670** |  |  |  |  |  |  |
| *Overall* | *ER-^*^* | *40-49* | 294 / 1240 | 1.03 (0.74-1.44) | 8.49x10^-1^ |  |
|  |  | *50-59* | 791 / 5000 | 1.13 (0.93-1.37) | 2.33x10^-1^ |  |
|  |  | *≥60* | 553 / 6390 | 1.00 (0.77-1.29) | 9.94x10^-1^ | 3.39x10^-1^ |
|  | *ER+^**^* | *<40* | 357 / 1499 | 0.91 (0.67-1.24) | 5.39x10^-1^ |  |
|  |  | *40-49* | 2715 / 2987 | 1.17 (1.02-1.34) | 2.84x10^-2^ |  |
|  |  | *50-59* | 5309 / 7892 | 1.16 (1.05-1.27) | 2.27x10^-3^ |  |
|  |  | *≥60* | 5940 / 10590 | 1.05 (0.96-1.15) | 2.90x10^-1^ | 1.62x10^-3^ |
| *Europeans* | *ER-^*^* | *40-49* | 290 / 1236 | 1.05 (0.75-1.47) | 7.71x10^-1^ |  |
|  |  | *50-59* | 787 / 4974 | 1.13 (0.93-1.37) | 2.25x10^-1^ |  |
|  |  | *≥60* | 553 / 6385 | 1.00 (0.77-1.29) | 9.97x10^-1^ | 4.58x10^-2^ |
|  | *ER+^**^* | *<40* | 357 / 1499 | 0.91 (0.67-1.24) | 5.39x10^-1^ |  |
|  |  | *40-49* | 2587 / 2876 | 1.17 (1.01-1.35) | 3.23x10^-2^ |  |
|  |  | *50-59* | 5286 / 7854 | 1.15 (1.05-1.27) | 3.28x10^-3^ |  |
|  |  | *≥60* | 5907 / 10565 | 1.05 (0.96-1.15) | 2.84x10^-1^ | 8.55x10^-3^ |

Results are presented overall and separately for Europeans in strata defined by age. Pooled analyses adjusted for study were performed. Estimates and p-values were not calculated where data were too sparse (< 100 cases and/or controls per category).

^*^ER-: estrogen receptor negative

^**^ER+: estrogen receptor positive

^a^Odds ratio per minor allele (A allele for rs2046210, G allele for rs12662670).

^b^P-value derived from a log-additive model.

^c^P-value for interaction between single nucleotide polymorphism and age-group.
