# Supplementary material for: Comparison of 6q25 Breast Cancer Hits from Asian and European Genome Wide Association Studies in the Breast Cancer Association Consortium (BCAC)
Source: PLoS One. 2012 Aug 7;7(8):e42380. doi: 10.1371/journal.pone.0042380 (PMC3413660; doi:10.1371/journal.pone.0042380)
Supplement: Table S6 — Association of rs2046210 and rs12662670 with breast cancer. (DOC) [file pone.0042380.s006.doc]

**Table S6: A**ssociation of rs2046210 and rs12662670 with breast cancer.

| **Estrogen receptor status** | **Number of cases/controls** | **OR (95% confidence interval)^a^** | **P-value^b^** | **P-heterogeneity^c^** |
| --- | --- | --- | --- | --- |
| **rs2046210** |  |  |  |  |
| *Overall* | 41,418 / 39,104 | 1.10 (1.07-1.12) | 3.17x10^-17^ |  |
| *ER-^*^* | 4867 / 30,548 | 1.18 (1.13-1.23) | 3.76x10^-12^ |  |
| *ER+^**^* | 18,910 / 34,529 | 1.07 (1.04-1.10) | 7.46x10^-6^ | 1.05x10^-4^ |
| **rs12662670** |  |  |  |  |
| *Overall* | 33,031 / 32,485 | 1.15 (1.11-1.20) | 2.62x10^-13^ |  |
| *ER-^*^* | 3858 / 24,314 | 1.15 (1.05-1.26) | 2.12x10^-3^ |  |
| *ER+^**^* | 16,233 / 28,191 | 1.10 (1.05-1.16) | 3.56x10^-4^ | 0.379 |

For these analyses, the study population was **restricted to those studies that did not oversample cases with a family history of the disease**, i.e. fourteen studies were excluded from the analyses. Results are presented overall as well as separately for estrogen receptor negative (ER-) and estrogen receptor positive (ER+) breast cancers. Pooled analyses adjusted for study were performed. A log-additive genetic model was assumed.

^*^ER-: estrogen receptor negative

^**^ER+: estrogen receptor positive

^a^Odds ratio per minor allele (A allele for rs2046210, G allele for rs12662670).

^b^P-value derived from a log-additive model.

^c^P-value for differences between main effects of single nucleotide polymorphisms in ER- versus ER+ cases derived from case-only analysis.
